# Can molecular dynamics simulations help in discriminating correct from erroneous protein 3D models?

**DOI:** 10.1186/1471-2105-9-6

**Published:** 2008-01-07

**Authors:** Jean-François Taly, Antoine Marin, Jean-François Gibrat

**Affiliations:** 1 INRA, Unité Mathématique Informatique et Génome UR1077, F-78350 Jouy-en-Josas, France

## Abstract

**Background:**

Recent approaches for predicting the three-dimensional (3D) structure of proteins such as *de novo *or fold recognition methods mostly rely on simplified energy potential functions and a reduced representation of the polypeptide chain. These simplifications facilitate the exploration of the protein conformational space but do not permit to capture entirely the subtle relationship that exists between the amino acid sequence and its native structure. It has been proposed that physics-based energy functions together with techniques for sampling the conformational space, e.g., Monte Carlo or molecular dynamics (MD) simulations, are better suited to the task of modelling proteins at higher resolutions than those of models obtained with the former type of methods. In this study we monitor different protein structural properties along MD trajectories to discriminate correct from erroneous models. These models are based on the sequence-structure alignments provided by our fold recognition method, FROST. We define correct models as being built from alignments of sequences with structures similar to their native structures and erroneous models from alignments of sequences with structures unrelated to their native structures.

**Results:**

For three test sequences whose native structures belong to the all-*α*, all-*β *and *αβ *classes we built a set of models intended to cover the whole spectrum: from a perfect model, i.e., the native structure, to a very poor model, i.e., a random alignment of the test sequence with a structure belonging to another structural class, including several intermediate models based on fold recognition alignments. We submitted these models to 11 ns of MD simulations at three different temperatures. We monitored along the corresponding trajectories the mean of the Root-Mean-Square deviations (RMSd) with respect to the initial conformation, the RMSd fluctuations, the number of conformation clusters, the evolution of secondary structures and the surface area of residues. None of these criteria alone is 100% efficient in discriminating correct from erroneous models. The mean RMSd, RMSd fluctuations, secondary structure and clustering of conformations show some false positives whereas the residue surface area criterion shows false negatives. However if we consider these criteria in combination it is straightforward to discriminate the two types of models.

**Conclusion:**

The ability of discriminating correct from erroneous models allows us to improve the specificity and sensitivity of our fold recognition method for a number of ambiguous cases.

## Background

The last 10 years have witnessed steady progress in the prediction of the three-dimensional (3D) structure of proteins from their amino acid sequence [1]. Most current approaches, ranging from *de novo *to fold recognition (threading) techniques, are based on simplified "energy" potentials (with a few exceptions such as the UNRES force field [2]). These empirical potentials, often more appropriately referred to as score functions, are derived from statistical analyses of structural features observed in known 3D structures: residue-wise interactions, secondary structure propensities, residue surface, etc. They are used with a reduced representation of the polypeptide chain (for instance, in many threading methods residues are represented by a single site, the C*β *or the C*α *atom). Such coarse-grained potentials cannot capture wholly the subtle relationship that exists between the amino acid sequence and the 3D structure of proteins. However their simplicity is an asset for exploring the conformational space, they permit to filter out improbable or unrealistic structures and, hopefully, to propose structures that are close to the native structure. *De novo *techniques, such as those developed by the Baker's group [3], generate hundreds of structures some close to the native structure but also many far from it. Threading methods are more constrained since they only consider known 3D structures and seek whether a particular query sequence might be compatible with some of these structures. Typically, they provide a list of template structures that are ranked by scores. All these methods have developed their own means to judge whether a *de novo *structure is close to the native structure or whether a particular template structure is compatible with a given sequence. However, in some cases this judgement is not clear-cut and it has been suggested that these techniques could benefit from the use of an atomic representation of the polypeptide chain together with detailed energy functions based on chemical-physical principles [4]. One would thus adopt a hierarchical approach, simplified potentials would permit to focus on a limited number of plausible candidates and detailed potentials would help in discriminating between native and misfolded structures. Since the pioneering work of Novotny *et al.*[5] a number of groups have sought to analyse whether usual molecular mechanics force fields, such as those employed for molecular dynamics (MD) simulations complemented with different ways of computing solvation effects, could indeed discriminate the native structure from misfolded conformations [6-13]. All these works, except [7] that uses a limited sampling, are based on minimised conformations, hence the need to introduce different terms to represent solvation free energy. It appears that such empirical free energy calculations are able to make a distinction, in many cases, between native structures and decoys. These works also analysed the role of the different terms involved in the free energy computation: internal, van der Waals and electrostatic energies, solvation contributions, etc. arriving at somewhat different conclusions as to which terms were preponderant for the discrimination.

Other groups [14-16] tackled a related question: can physics-based potentials together with an extensive sampling of the protein conformational space using MD simulations refine, i.e., bring closer to the native structure, an initial model resulting from a previous stage employing coarse-grained potentials?

In this work we wish to answer a very pragmatic question. We have developed a fold recognition method called FROST [17]. In this method we perform a statistical analysis of the scores to decide whether a sequence is compatible with a template structure. This permits us to recover 60% of the related sequence-structure pairs while keeping an error rate of 1%. Unfortunately 40% of the pairs do not satisfy this statistical criterion although, for an important fraction of them, a compatible template structure can be found amongst the top templates of the list ordered by normalised scores. Hereafter, we will refer to the zone where scores are not statistically significant as the "twilight zone" of the method. The question we would like to answer is thus the following: can physics-based potentials and MD simulations help us in discriminating "correct" from "erroneous" templates in such borderline cases. Our motivation is similar to the one of Kinjo and colleagues [11] although, as described below, we employ a different approach.

We define a correct template as a structure that is similar to the query sequence native structure and an erroneous template as any other structure of the database. Let us note that these erroneous templates, because they appear in the top of the list, constitute difficult cases compared to what has been done by Novotny *et al.*[5]. They just swapped the sequences between an all-*α *protein and an all-*β *protein without paying attention whether this procedure buried charged residues or exposed non polar residues. The threading method will find the best location for the query residues in the 3D structure according to its empirical score function (the current version of the method insures that the best alignment score between the query sequence and the template structures is found). Therefore many of the top erroneous templates are likely to exhibit some common features with the correct templates making them difficult to distinguish.

To answer the above question, for the borderline cases, we build 3D models based on the sequence-structure alignments provided by our fold recognition method and we submit them to MD simulations. We monitor different structural features: RMSd, radius of gyration, secondary structures, side chain surface, etc. along the trajectories to discriminate correct from erroneous models. There are many ways in which a 3D model can be erroneous. In this study, to be consistent with the question we want to answer, an erroneous model is defined as a model for which the sequence has been aligned with a template structure that has no relationship with its native structure.

Notice that, in real cases, one cannot use approaches similar to those described in [6-13] since they require the knowledge of the native structure. In these studies, the value of the free energy for the native structure is usually smaller than the value of the free energy for the decoys but the difference is often quite small relative to the magnitude of the figures involved (a few percents at best). Free energy values are very different according to the protein studied and there is no absolute scale on which one could rely. Therefore instead of trying to compute the free energy of the models we choose to rely on the analysis of the behaviour of different structural features along the MD trajectories.

## Results

We selected 3 query domains representative of the principal SCOP [18] classes: all-*α*, all-*β *and *αβ*. For each query domain we built 6 models as described in the Method section (listed in Table 1). These models correspond to the *same sequence *(the query sequence) aligned with different template structures. They cover the whole spectrum of models that can be generated for a query sequence: from a perfect model corresponding to the native structure (NS0) to a very poor model where the query sequence has been aligned with a template structure completely different from its native structure (DT2).

**Table 1:** Summary of the characteristics of the 6 models built for each query structure

**Model**	**Characteristics**		
	Template	Alignment	% seq. Id.
NS0	Native struct.	Exact	100
ST1	Similar	Good	20 – 30
ST2	Similar	Fair	0 – 20
ST3	Similar	Poor	0 – 15
DT1	Different	-	0 – 15
DT2	Different	-	0 – 15

As a mnemonic, the first letter indicates whether the query sequence has been aligned with the right template (*S*) or not (*D*) and the last digit indicates the proximity of the model with the true structure. (See method section for further detail).

**Figure 1:**
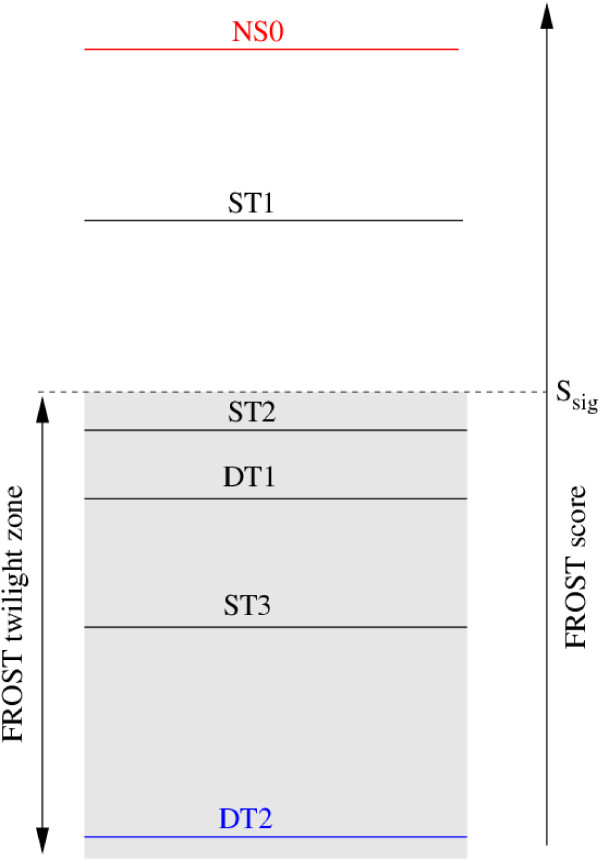
Schematic representation of the ranking, according to the FROST score, of the template structures that are used to build the 6 models for a given query sequence (template structures are designated by the corresponding model name). *S*_*sig *_represents the score above which there is less than 1% chance to find a false positive [17]. Below this score we define the "twilight zone" of the method. In this zone the score does not allow us to predict with enough confidence whether the template structure is similar to the query sequence structure or not. DT1 is the first false positive, i.e., the template structure, not similar with the query structure, which has the highest FROST score. In this work the template structures we chose for ST2, DT1 and ST3 have comparable scores and they are found in the twilight zone. The template structure for ST1 has a score that is usually above *S*_*sig*_. The ordering of the template structures for ST2, DT1 and ST3 can change from what is depicted on the figure depending on the query sequence.

Between these two extremes, that are used as controls, we wish to focus on intermediate models that are typical of what can be expected after a fold recognition analysis (see Fig. 1). For the first three intermediate models, ST1, ST2, ST3, the query sequence has been aligned with a template structure that is similar to the native structure. For ST1 and ST2 the alignment is good or fair (see Additional file 1, Tables S1 and S2 for a precise definition of these terms). These two models are mainly distinguished by the percentage of sequence identity between the query sequence and the sequence of the template structure: ST1 is in the range 20–30 approaching the generally recognised limit of 30% for homology modelling [19]. ST2 has been chosen, on purpose, to lie within the twilight zone. Usually this implies that the percentage of sequence identity is below 20%, typical of fold recognition targets. ST3 has the same characteristics as ST2 except that we selected a template structure on which the fold recognition method was not able to align correctly the query sequence. This is often the case when the template structure, although globally similar to the query native structure, possesses additional secondary structure elements or when secondary structure elements of the "core" have undergone substantial motions relative to each other. These two characteristics perturb the fold recognition alignment. The last model, DT1, has been obtained by aligning the query sequence with a template structure that is different from its native structure. It must be noticed that we chose the template structure that corresponds to the first false positive appearing in the fold recognition list of structures ranked by score. Therefore, according to the FROST score function, this template structure possesses some features similar to the query native structure. Indeed we often observe that such template structures share motifs with the query native structure that tend to confuse the fold recognition method.

Let us emphasise that all models, as far as the backbone is concerned, are based on known 3D structures. Structural features such as the network of hydrogen bonds, the Ramachandran zones for the *φ *and *ψ *angles, etc. are realistic (with the possible exception of regions corresponding to the loops built by Modeller). The only unrealistic structural feature concerns the location and interactions of side chains in some of the models.

In the following, we try, by monitoring various structural characteristics along MD trajectories at different temperatures, to answer two questions. First, is it possible to distinguish, quantitatively or qualitatively, models for which the query sequence has been aligned with a template structure similar to its native structure from models for which the query sequence has been aligned with template structures without relation with its native structure? If the query sequence has been aligned with the correct template structure but with a poor alignment can we pinpoint parts of the model that correspond to misaligned regions?

### Analysis of the models

#### All-*α *models

**Figure 2:**
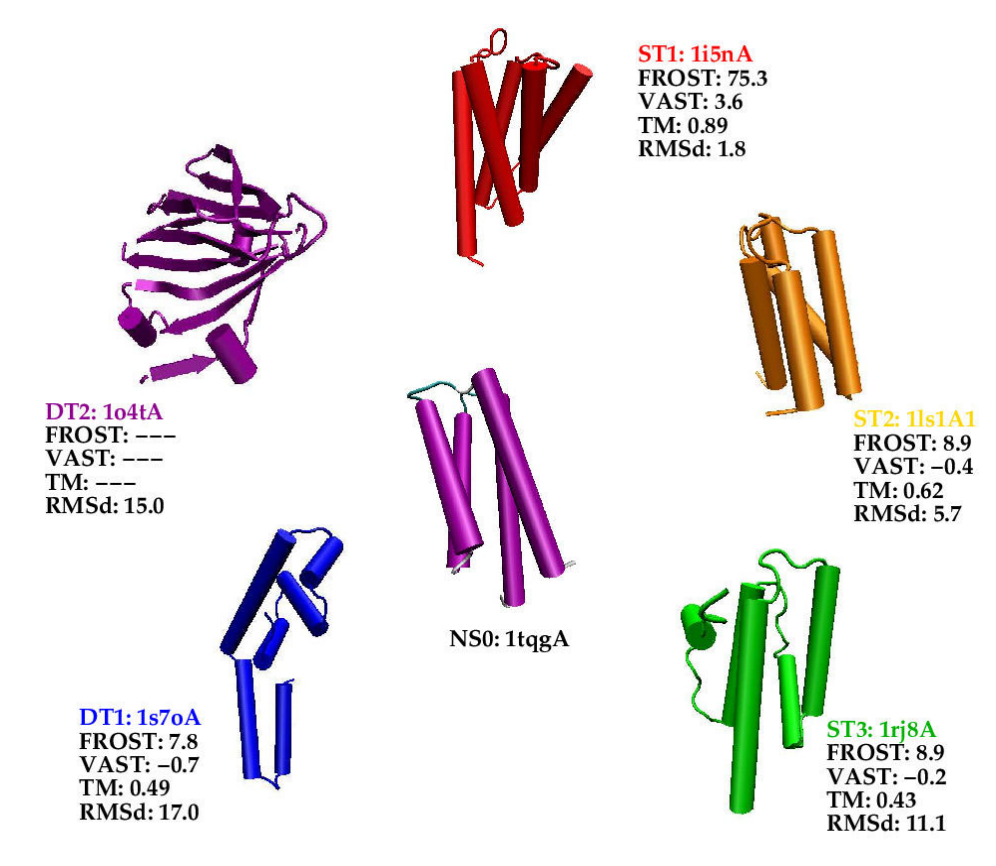
1tgqA (labelled NS0), is the query protein that was selected for the all-*α *class. The first 4 characters correspond to the PDB code, the uppercase letter to the chain name and the last digit to the SCOP domain number, if any. The template structures that are used for the models are shown clockwise (they are labelled with the corresponding model name). FROST: is the FROST score. *S*_*sig *_that defines the beginning of the twilight zone (see Fig. 1) is equal to 15. VAST: is the VAST score. A VAST score greater than 2 is indicative of a clear structural similarity. Below this value the structural relationship is unclear. TM: is the TM-score value. A value larger than 0.4 indicates that the proteins are structurally related, a value less than 0.17 that they are not related and a value in between corresponds to a zone were the structural relationship between the proteins is ambiguous. RMSd: is the Root Mean Square displacement value in Å between the corresponding *model *and the query native structure.

Fig. 2 shows the query protein for the all-*α *class and the template structures that are used to build the models. In Additional file 1, Table S1 gives details about the structure comparisons, Figure S1 displays the relationship that exists between these structures according to the SCOP hierarchy, Table S2 shows comparisons of the models with the query native structure and Figure S2 shows the structural superimposition of the models with the query native structure. Notice that not all models have a length equal to the query sequence length (105 residues). Modeller does not know how to handle properly residues that, according to the alignment, are located before the N-terminus, or after the C-terminus of the template structure. In such cases it generates extended conformations. We thus decided to chop these residues in the alignment.

Fig. 2 shows the FROST score (the twilight zone lies below *S*_*sig *_= 15), the VAST score (a score above 2 is indicative of a clear structural relationship between the proteins), the TM-score (a score larger than 0.4 implies a structural relationship between the proteins) and the RMSd of *the model *with respect to the query native structure.

The FROST score is very high for model ST1 since this model is in the range of homology modelling. Scores for other models are in the FROST twilight zone with a small difference between scores for ST2, ST3 and DT1. This is precisely to help us resolving such ambiguous situations that we would like to employ MD simulations.

All indicators show that ST1 is related to the query native structure. For ST2 the VAST score is negative but the TM-score is significant. ST3 and DT1 appear to have about the same weak similarity with the query structure. The TM-score is slightly above the significant threshold of 0.4. In fact, according to SCOP, ST3 belongs to the same fold as the query protein (4-helical up-and-down bundle) whereas DT1 belongs to a different fold (DNA/RNA-binding 3-helical bundle). These two folds obviously share a common helical motif that confuses the fold recognition program.

Model ST2 is marginally similar to the query native structure with an RMSd of 5.7 Å

#### All-*β *models

**Figure 3:**
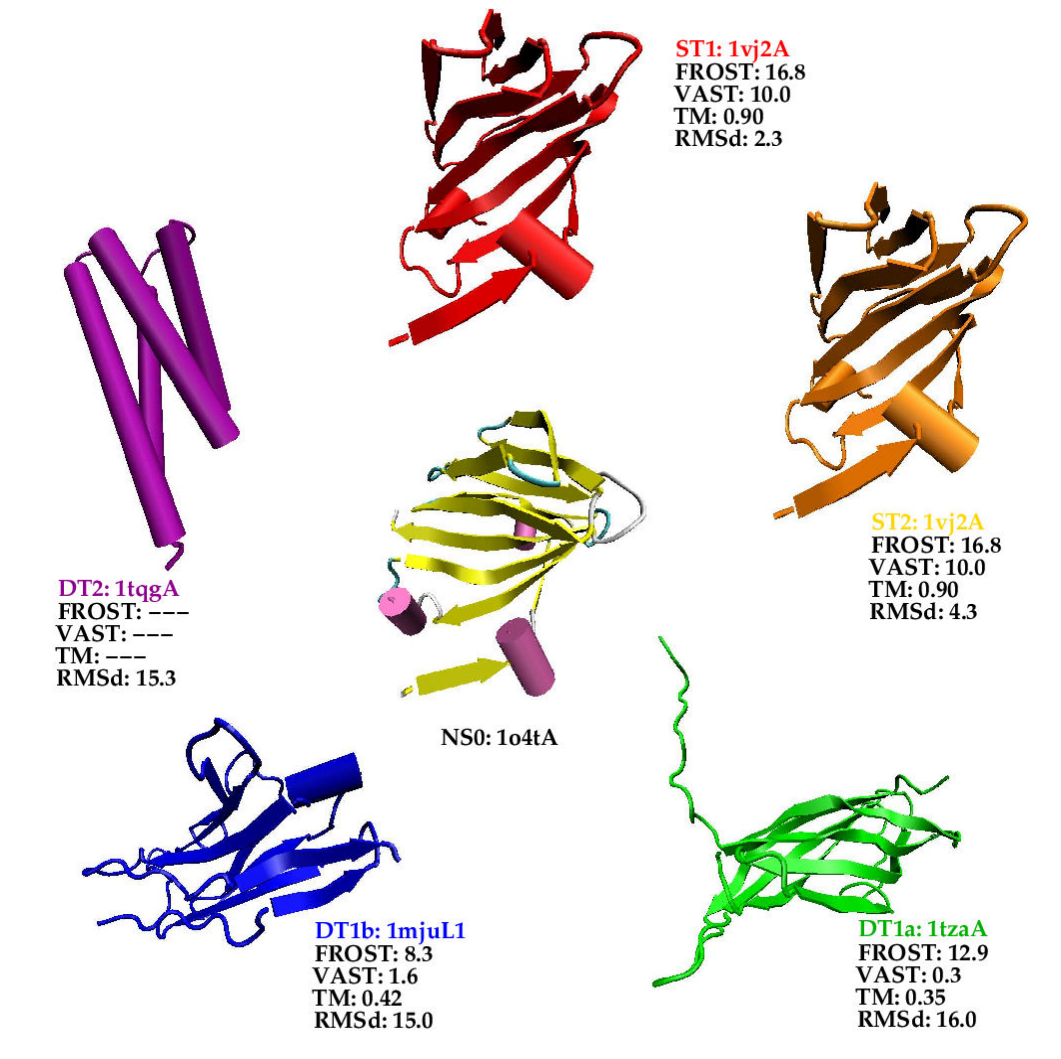
Similar figure as Fig. 2 for the all-*β *class.

Fig. 3 shows the query protein for the all-*β *class and the template structures that are used to build the models. In Additional file 1, details about the structure comparisons are given in Table S1, Figure S3 displays the relationship that exists between these structures according to the SCOP hierarchy, Table S2 shows comparisons of the models with the query native structure and Figure S4 shows the structural superimposition of the models with the query native structure.

Notice that the all-*β *query structure, 1o4tA, has been used as template to build the DT2 model for the all-*α *class. Conversely, the all-*α *query, 1tqgA, is used to build the DT2 model for the all-*β *class.

For this class it was difficult to find templates for the whole range of models. For instance we could not find a template for model ST1. Therefore we decided that the template for ST2 would also be used as template for ST1. The two models just differ by the type of alignment that is used: ST1 is based on the structural alignment whereas ST2 is based on the fold recognition alignment. We could not find either a template for the ST3 model. Instead we built two DT1 models, named DT1a and DT1b. The templates used for DT1a and DT1b belongs to the same SCOP fold (Immunoglobulin-like *β *sandwich) but to different super-families.

The template used for models ST1 and ST2 is clearly similar to the query structure. Both this template and the query structure belong to the same SCOP family of the double-stranded *β *helix SCOP fold. The query protein, 1o4tA, whose length is 115 residues, is a homodimer in the PDB file. At its N-terminus there exists a small strand that pairs with its counterpart in the other monomer. When using only the monomer this strand is floating in the solvent. We thus decided to remove this portion of the sequence (9 residues) from the NS0 model to obtain a more compact 3D structure. However alignments with template structures where performed with the complete sequence. This explains why the DT1b model is 112 residues long.

#### *αβ *models

**Figure 4:**
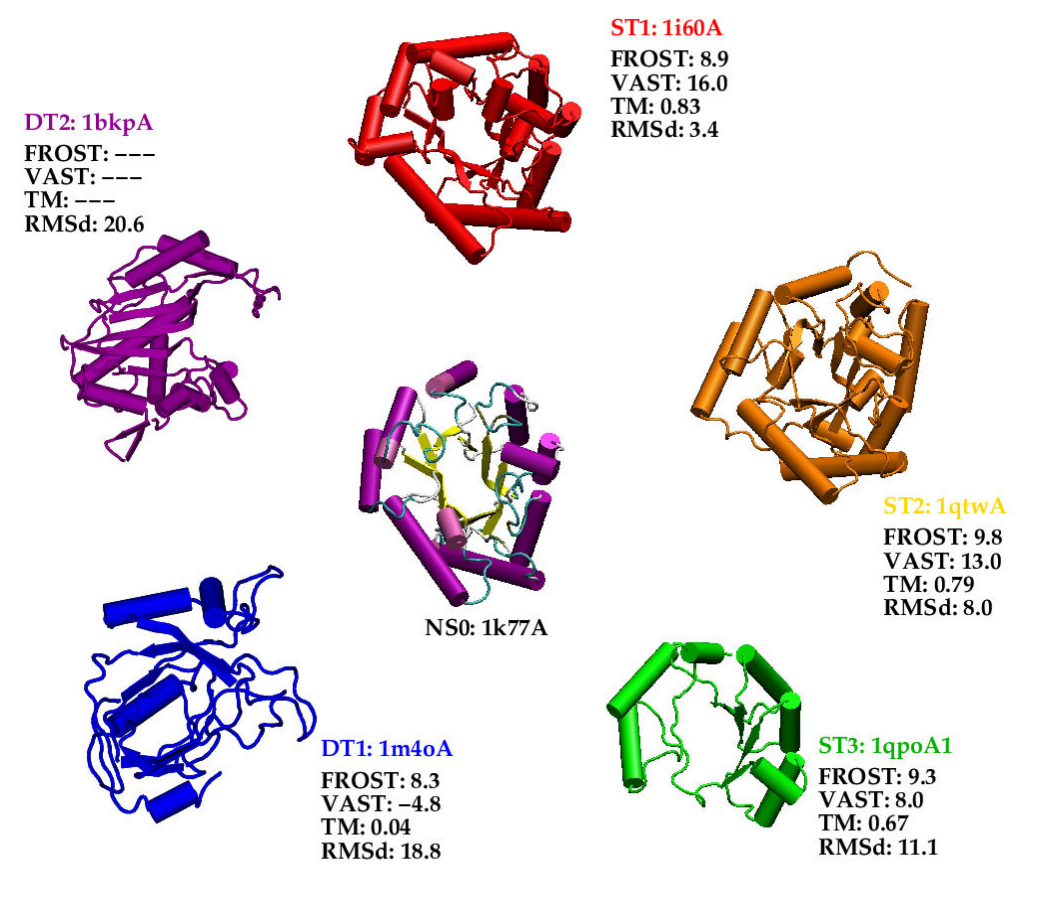
Similar figure as Fig. 2 for the *αβ *class.

Fig. 4 shows the query protein for the *αβ *class and the template structures that are used to build the models. In Additional file 1, Table S1 gives details about the structure comparisons, Figure S5 displays the relationship that exists between these structures according to the SCOP hierarchy, Table S2 shows comparisons of the models with the query native structure and Figure S6 shows the structural superimposition of the models with the query native structure.

For the *αβ *class it was easier to find template structures that span the whole range of models. Structural indicators reveal that the ST models are clearly related to the query structure. FROST scores are in the twilight zone and it is difficult to differentiate ST and DT template structures. Overall the percentage of sequence identity between the template proteins and the query protein is weak (at most 18% for the ST1 template structure). Accordingly the FROST score for ST1 is within the twilight zone. ST3 template structure, 1qpoA1, corresponds to a domain of a larger protein. This domain corresponds to three-quarter of a regular TIM-barrel: the inner *β *barrel has only 6 strands and is not closed; there are 6 external *α *helices.

The ST1 model is close to the query structure whereas the ST2 model is relatively far from it (RMSd = 8.0Å). DT models are far from the query structure. ST3 model is intermediate between DT and the two other ST models.

The template structure that is used to build the DT2 model, 1bkpA, belongs to the SCOP *α *+ *β *class.

### Conformational space sampling efficiency

We have carried out a principal component analysis as described in the Method section. This analysis is usually employed to study collective motions of the system that are described by the first few principal modes. Here we use it, primarily, to check whether the sampling of the conformational space we performed is sufficient, through the computation of the cosine content of the first 3 principal components.

It has been shown that the principal components of random diffusion are cosines with the number of periods equal to half the principal component index [20,21]. When the cosine content of the first few principal components is close to 1, the largest fluctuations are not connected with the potential but with random diffusion. In other words, the corresponding simulation was not long enough for the system to sample the "walls" of the potential energy function.

The maximum cosine content observed for all the models is 0.65 (data not shown). Only *αβ *models consistently exhibit values close to 0.65 for one of the first 3 modes, other models have lower cosine content. From these results one can conclude that the sampling was sufficient to explore the local minimum region for all-*α *and all-*β *models. For *αβ *models, the length of the simulation might possibly be a bit too short, although it is difficult to interpret this value of 0.65 with certainty. Two dimensional plots showing the projection of the trajectories along pairs of modes confirm globally these conclusions (data not shown). Let us emphasise that a low cosine content is not a proof that the system has *exhaustively *explored all the conformational space available to it at a given temperature. In our experience, with other systems, the cosine content can be low for the first 4 ns, say, then can rise suddenly when we compute it for the first 6 ns. The interpretation of this phenomenon is that the system got trapped inside a region of the conformational space during the first 4 ns then managed to overcome a barrier of potential after 4 ns to reach a new region. After 6 ns the system has not had time, yet, to sample the boundaries of this new region. Therefore if the cosine content computed for a given trajectory is close to 1 we know that the corresponding simulation was not long enough to explore the local conformation space completely. On the other hand, if the cosine content is low, we know that the system has had enough time to explore the local potential valley but we cannot be sure that, had we continued the simulation further, the system would not have reached, eventually, another valley.

### Root Mean Square deviation analysis

To analyse the behaviour of the different models along the molecular dynamics trajectories we monitored the root-mean-square deviation (RMSd) between the initial conformation of the production phase (t = 0) and conformations taken every 10 ps. For this criterion, as well as for the other criteria we consider below, we seek i) whether there exists a correlation between the spectrum of models and the criterion values and ii) whether we can find a value of the criterion that allows us to clearly discriminate ST models from DT models.

Table 2 shows the average RMSd values, standard deviations at three temperatures (300 K, 350 K and 400 K) for the models of the all-*α*, all-*β *and *αβ *classes.

**Table 2:** Root Mean Square deviations

		300 K		350 K		400 K	
		
Class	Models	Mean	sd	Mean	sd	Mean	sd
all-*α*	NS0	1.3	0.21	1.8	0.38	2.3	0.43
	ST1	2.2	0.31	2.2	0.31	3.0	0.56
	ST2	2.8	0.32	3.4	0.46	4.6	0.65
	ST3	3.6	0.48	3.9	0.55	5.3	1.17
	DT1	5.8	0.80	6.2	1.19	9.8	2.17
	DT2	2.7	0.45	3.5	0.55	4.8	0.92

all-*β*	NS0	1.9	0.19	2.2	0.26	2.2	0.25
	ST1	2.4	0.29	2.2	0.29	2.9	0.30
	ST2	2.3	0.27	2.7	0.53	3.6	0.52
	DT1b	2.9	0.28	3.5	0.42	4.4	0.45
	DT1a	3.7	0.50	4.0	0.49	5.5	0.97
	DT2	2.8	0.41	4.1	0.89	6.5	1.22

*αβ*	NS0	1.6	0.22	2.2	0.25	2.9	0.37
	ST1	2.6	0.26	3.3	0.38	3.6	0.36
	ST2	3.3	0.33	4.2	0.40	5.2	0.60
	ST3	3.4	0.31	5.0	0.48	5.0	0.52
	DT1	4.4	0.68	6.2	0.61	7.7	0.79
	DT2	3.6	0.50	4.3	0.52	5.9	0.70

For each models, RMSd between the initial conformation of the trajectory and conformations taken every 10 ps along the trajectory at different temperatures. mean: average value along the trajectory, sd: standard deviation. All figures are in Å

Before analysing Table 2, we must first determine whether the average and standard deviation values listed in these tables are significantly different. For this we performed an analysis using Student t- and F-tests. These tests compute the probability (p-value) that two distributions have the same means and variances. These computations critically depend on the number of data points. It is also assumed that these points are independent. For molecular simulations trajectories the latter property can be questioned. To evaluate the effect of correlations between conformations along the trajectory we computed the same p-values with only one tenth of the points, that is, conformations separated by 100 ps corresponding to 50,000 molecular dynamics steps. Results of these computations are given in Additional file 1, Fig. S7. According to this analysis the observed differences in the means are always significant. For the variances the differences are most of the time significant except for the all-*α *model pairs ST1-ST2 and DT2-ST3, the all-*β *model pairs DT1b-ST1 and DT1b-ST2, and the *αβ *model pair ST2-ST3.

Inspection of Table 2 for trajectories at 300 K reveals a rough correlation between the model accuracy along the spectrum of models and the RMSd mean or the standard deviation (that measures the fluctuations of the molecule along the trajectory). With the notable exception of DT2 models, the mean and standard deviation steadily increase when one goes from NS0 to DT1 models.

DT2 models do not behave as expected. Their RMSd mean is close to the values of the ST1-2 models. However their standard deviation is in the range observed for DT1 models, clearly above the standard deviations of ST1-2 models. We will see that, for other criteria too, DT2 models display an anomalous behaviour. We will delay until the end of the Result section the analysis of DT2 models to explain this unexpected behaviour.

Performing simulations at different temperatures does not help in discriminating the different types of models. The RMSd mean and standard deviation increase with the temperature but approximately in the same proportion for all the models. Only the all-*β *DT2 model exhibits the behaviour we were expecting. Its RMSd mean increases by a factor of more than 2 and its standard deviation by a factor of 3 between 300 K and 400 K. These figures are clearly larger than those observed for other models.

### Analysis of conformation clusters

To further characterise the behaviour of the models along the trajectories we minimised the previous conformations in vacuum and clustered the resulting minimum conformations as described in the Method section. In this way we seek to obtain a coarse estimate of the number of conformational space regions explored by the different models along the trajectories. Table 3 shows the number of clusters for all models of the 3 classes as a function of the simulation temperature. Cut-off values for the clustering procedure have been chosen such as to result in a single cluster for NS0 models at 300 K. The choice of single linkage to generate the clusters is deliberate. With this method, we expect all the conformations that are in the same potential energy valley to cluster together. Even though some of them might be farther apart than the cut-off value, it should always be possible to find intermediate conformations that link them. On the contrary, when the system crosses a sufficiently high energy barrier (which thus constitutes a rare event during the length of the simulation) to reach a new potential energy valley it should be less likely to find such intermediate conformations and two clusters should be obtained (of course this is true only because we consider conformations that are 10 ps apart along the trajectory).

**Table 3:** Number of clusters as a function of the simulation temperature

Class	Model	300 K	350 K	400 K
all-*α*	NS0	1	1	70
	ST1	2	16	192
	ST2	4	193	711
	ST3	54	377	879
	DT1	1045	1059	1091
	DT2	98	931	1060

all-*β*	NS0	1	1	1
	ST1	1	1	5
	ST2	1	1	7
	DT1b	2	23	152
	DT1a	109	34	298
	DT2	1	18	41

*αβ*	NS0	1	1	1
	ST1	2	2	2
	ST2	9	29	29
	ST3	10	41	199
	DT1	495	198	383
	DT2	55	65	225

Results shown in Table 3 allow a better discrimination between the different types of models than results presented in Table 2.

We observe the same general correlation between the spectrum of models and the number of clusters. DT2 models still behave as outliers but there exists a better contrast with respect to ST1-2 models than previously (except for the all-*β *class). At 300 K NS0 and ST1-2 models for classes all-*α *and *αβ *explore at most 10 clusters whereas DT2 models explore several tens. Increasing the temperature, in particular in the all-*β *case, does improve the discrimination between the model types. For all-*β *DT1b and DT2 models, that are indistinguishable from NS0 and ST1-2 models at 300 K, the number of explored clusters increases steadily with the temperature whilst it remains small for NS0 and ST1-2 models. As previously, ST3 models are intermediate between ST1-2 and DT models although the all-*α *ST3 model seems closer to DT models and the *αβ *ST3 model to ST1-2 models.

### Evolution of the secondary structure content along the trajectories

We monitored the secondary structures along the MD trajectories for the different models. We used the Gromacs tool do_dssp that encapsulates the secondary structure assignment program dssp [22]. This program assigns the secondary structures based on the existence of hydrogen bonds between backbone donor and acceptor atoms. Since hydrogen bonds are relatively sensitive to atomic fluctuations we decided to calculate the secondary structures for minimised conformations along the trajectories. Table 4 shows the fraction of residues that are part of a periodic secondary structure (*α *helix or *β *strand) in the initial conformation and that remain in this secondary structure for at least 95% of the minimized conformations along the trajectory.

**Table 4:** Percentage of secondary structures

Class	Model	300 K	350 K	400 K
all-*α*	NS0	96	90	81
	ST1	87	85	74
	ST2	90	61	45
	ST3	71	50	24
	DT1	54	47	13
	DT2	38	13	10

all-*β*	NS0	69	61	55
	ST1	55	58	47
	ST2	41	40	34
	DT1b	59	52	26
	DT1a	11	20	9
	DT2	87	67	38

*αβ*	NS0	80	73	65
	ST1	68	51	48
	ST2	52	33	16
	ST3	52	43	37
	DT1	41	27	0
	DT2	44	31	8

Percentage of residues having a periodic secondary structure (*α *helix or *β *strand) in the trajectory initial conformation that keep this secondary structure in 95% of the trajectory conformations. Conformations along the trajectories have been minimised in vacuum.

The first point to stress is that *α *helices and *β *strands do not display the same stability [23]. Considering NS0 models for the different classes, it appears that *α *helices are more persistent than *β *strands.

This property seems to have an impact on the relevance of the secondary structure as a criterion for discriminating the different types of models. For all-*α *models there is a good correlation between the percentage of secondary structures kept along the trajectory and the different types of models, even for the DT2 model that behaved as an outlier for the RMSd criteria we examined above. This is true for all temperatures. The secondary structure criterion allows a good distinction between DT and ST models, including the ST3 model that showed a tendency to behave as DT models with RMSd criteria.

For *αβ *models there is still a correlation between the percentage of conserved secondary structures and the type of models although this correlation becomes less regular when the temperature increases. It is more difficult to discriminate DT and ST models.

For the all-*β *class, DT1b and DT2 models perturb the correlation. The case of the DT2 model appears surprising at first sight since it exhibits a higher fraction of conserved secondary structures than the native structure itself at 300 K. In this model the native sequence has been aligned with the all-*α *query structure. This confirms our previous observation that *α *helices appear to be more stable than *β *strands. Let us note, however, that the NS0 model looses few secondary structures when the temperature increases unlike the DT2 model whose fraction of secondary structures is halved when the temperature is raised from 300 K to 400 K. For this class the secondary structure criterion is ineffective in discriminating the different types of models.

### Statistical score based on residue surface areas

The accessible surface area of the residues is an important parameter to characterise protein 3D structures. To quantify this parameter we defined an empirical score function based on the residue surface areas. This score function takes into consideration the so-called "polar satisfaction" of the polar residues and the "hydrophobic satisfaction" of hydrophobic residues. These two criteria are intended to account for the observed tendency of hydrophobic residues to be buried and polar residues to be in contact with the solvent or other polar residues. The method section briefly describes how this score function is calculated. We compute 2 types of scores. The first one is a global score for the whole protein, the second is a window score that is the mean of the scores in an 11-residue window centred on a residue of interest (each residue in the sequence is thus characterised by a particular window score).

Table 5 shows the global score at the beginning of the MD simulations and the mean global scores along trajectories at 300, 350 and 400 K for the different models of the three above classes. The scores are computed on the minimised conformations along the trajectories. The initial conformation at t = 0 is also minimised before computing its score. In Table 5 figures in italic are beyond the value characteristic of the 1% confidence threshold. For instance, for real 3D structures of the all-*α *class only 1% of the proteins are expected to have a global score less than -1.29 (see Additional file 1, Table S3). All-*α *models whose global score is less than this value are considered to differ significantly (with a 1% confidence level) from a real 3D structure.

**Table 5:** Global score based on residue surface areas

Class	Model	*S*_*t *= 0_	$\bar{S}$ 300 K	$\bar{S}$ 350 K	$\bar{S}$ 400 K
all-*α*	NS0	**-0.96**	**-0.87**	**-0.87**	**-0.91**
	ST1	**-1.12**	**-0.86**	**-0.89**	**-0.92**
	ST2	*-1.42*	**-1.16**	**-1.27**	**-1.23**
	ST3	*-1.70*	*-1.49*	*-1.52*	*-1.44*
	DT1	*-1.73*	*-1.48*	*-1.34*	*-1.37*
	DT2	*-1.88*	*-1.68*	*-1.66*	*-1.67*

all-*β*	NS0	**-1.17**	**-1.04**	**-1.07**	**-1.07**
	ST1	**-1.29**	**-1.13**	**-1.13**	**-1.12**
	ST2	*-1.47*	**-1.22**	**-1.19**	**-1.23**
	DT1b	*-1.77*	*-1.66*	*-1.54*	*-1.41*
	DT1a	*-1.92*	*-1.68*	*-1.66*	*-1.61*
	DT2	*-1.87*	*-1.65*	*-1.63*	*-1.55*

*αβ*	NS0	**-1.11**	**-1.00**	**-1.03**	**-1.06**
	ST1	*-1.25*	**-1.14**	**-1.13**	**-1.10**
	ST2	*-1.60*	*-1.47*	*-1.45*	*-1.44*
	ST3	*-1.73*	*-1.47*	*-1.39*	*-1.43*
	DT1	*-1.75*	*-1.49*	*-1.42*	*-1.44*
	DT2	*-1.68*	*-1.58*	*-1.45*	*-1.50*

*S*_*t *= 0 _is the score of the initial conformation; $\bar{S}$ is the mean score averaged over trajectories, for different temperatures. Scores in italic are beyond the value characteristic of the 1% confidence interval (see Additional file 1, Table S3)

It is interesting to notice that MD simulations, although the physics-based function they employ does not take into account the residue surface areas, systematically improve the global score for all the models with respect to the static models at t = 0. In particular this allows the all-*α *and all-*β *ST2 models and the *αβ *ST1 model, that appear significantly different from a true structure when only the static initial conformation at t = 0 is considered, to be brought within the range of scores characteristic of real 3D structures. The improvement for NS0 models is, in general, less than the improvement observed for other models. The worst models show a tendency to exhibit the largest score improvements, although this is not systematic.

The global score allows the discrimination of the ST1-2 models (except for the *αβ *class). ST3 models behave as the DT models with this criterion. The scores of DT models are usually far below the 1% confidence threshold value (several standard deviations). DT models are thus easy to detect.

**Figure 5:**
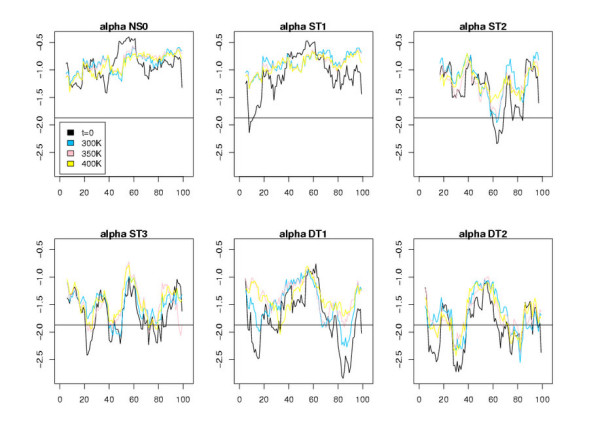
Plots of the window scores for the residues of the different all-*α *models. The x-axis corresponds to the residue index and the y-axis to the score values averaged over the MD trajectory (except for t = 0). The horizontal line represents the 1% confidence interval value, i.e., the value beyond which there is less than 1% chance to find a window score when it is calculated with observed 3D structures.

**Figure 6:**
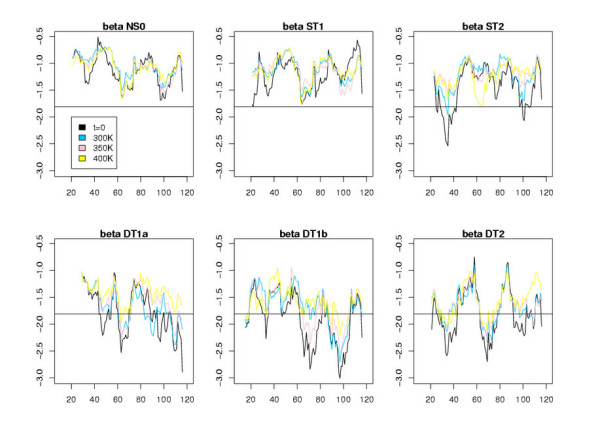
Same plots as those shown in Fig. 5 for all-*β *models.

**Figure 7:**
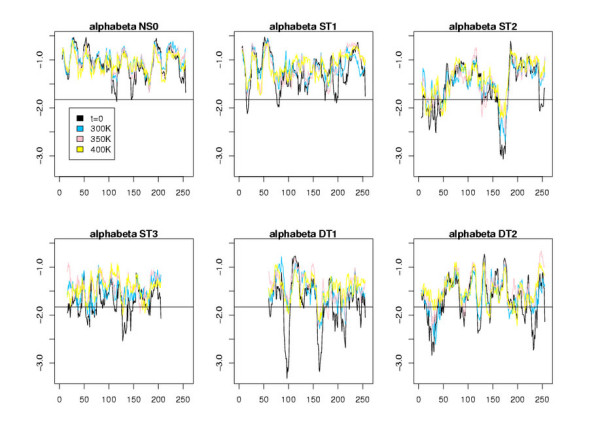
Same plots as those shown in Fig. 5 for *αβ *models.

Figures 5, 6, 7 display the window scores for the same models. The horizontal black line is the value corresponding to the 1% confidence threshold for the window score (see Additional file 1, Table S3). The black curves represent the window scores for the initial conformations, the three other curves the mean of the window scores at different temperatures. We observe that, except for NS0 models, all models display zones with window scores below the 1% confidence threshold. These zones of the models differ significantly from true 3D structure in terms of surface area of hydrophobic/hydrophilic residues. As expected, the number of peaks that cross the 1% confident threshold increases along the model spectrum. ST1 models show few such peaks. For ST2 models there are at least a couple of zones with very low scores. ST3 models, visually, are difficult to distinguish from DT models. These observations, of course, are consistent with results shown in Table 5. The principal benefit of the window score over the global score is that it allows us to single out regions of the models that are likely wrong. We thus investigated further, for different models, those peaks that show a clear improvement of the initial conformation at t = 0 during the MD trajectory.

**Figure 8:**
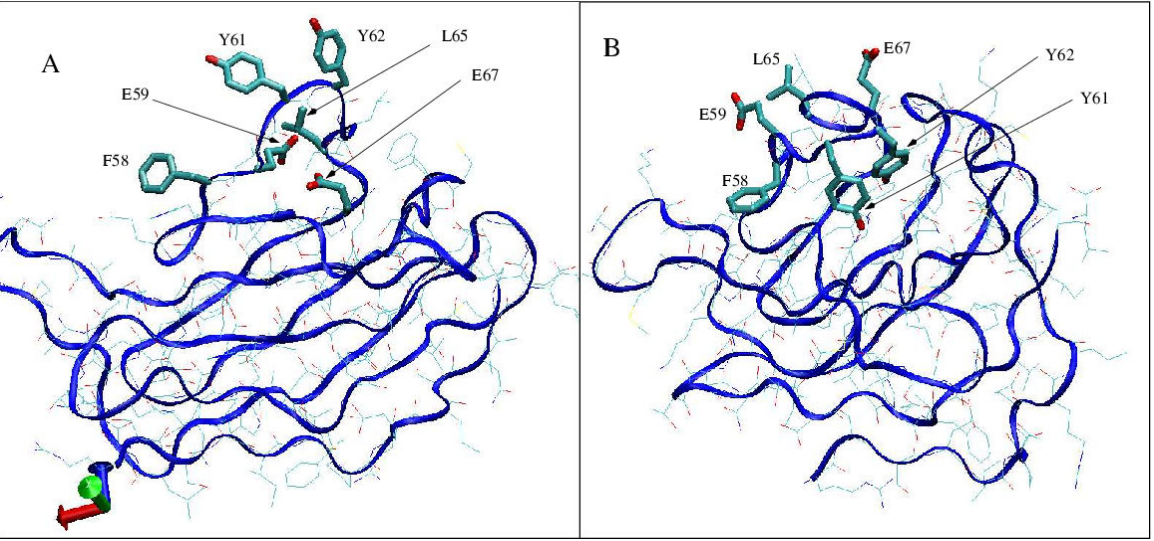
Schematic representation of the all-*β *DT1a model. The top panel (A) corresponds to the initial conformation at t = 0 after minimisation in vacuum. The bottom panel (B) corresponds to the conformation at t = 280 ps (also minimised in vacuum). The backbone trace is shown as a blue ribbon, most residues as thin lines. Some residues of interest (see Table 6) are drawn with a "liquorice" representation. Oxygen atoms are coloured red, nitrogen atoms dark blue, carbon atoms cyan. This figure was created with VMD [37].

To illustrate these investigations we describe in Table 6 and Figure 8 the case of the region centred at residue Tyr 62 of the all-*β *DT1b model. This region corresponds to the second lowest peak, at position 62, in Fig. 6. We chose this region because i) it exhibits a large improvement between the window score of the initial conformation and the mean value for the trajectories at 400 K and 300 K and ii) it is representative of what we observed for other such regions. Table 6 shows the scores for the aforementioned region. Both individual residue scores and window scores are shown. The first two lines correspond to the initial conformation, at t = 0 ps. The next two lines represent the same scores for a snapshot of the MD trajectory at 400 K. The selected snapshot is the one for which the value of the individual score of Tyr 62 is minimum. The last 2 lines once again display the same scores but this time for the residues in their native structure, for the purpose of comparison. Residues in bold are those with individual scores less than -2.8. Their side-chains are shown in Fig. 8 with a "liquorice" representation, other residues being displayed as thin lines and the trace of the backbone as a blue ribbon. Oxygen atoms are coloured red, nitrogen atoms in dark blue and carbon atoms in cyan. Fig. 8-A shows the reasons why these residues have such poor scores. Hydrophobic residues: Phe 58, Leu 65, Tyr 61 and Tyr 62 point directly to the solvent. On the contrary, polar residues Glu 59 and Glu 67 are buried in the interior of the structure. Notice that, although tyrosines have a polar oxygen, they are considered as hydrophobic residues in this analysis. The reason is that, in native 3D structures, their oxygen usually manages to point to the solvent or to interact with another polar atom whereas the rest of the side-chain remains buried among non polar atoms. Fig. 8-B shows the same residues after 280 ps along the trajectory. The two glutamates now point toward the solvent and the hydrophobic residues show a tendency to be buried inside the 3D structure. Tyr 62 succeeds in creating a hydrogen-bond with an acceptor atom inside the structure unlike Tyr 61. This might explain why Tyr 61 side-chain is only partially buried. In the native 3D structure this region of the sequence, from Phe 58 to Leu 64, corresponds to a *β *strand that is preceded and followed by turns. In the native structure Glu 59 is buried inside the structure, which explains its unfavourable score of -5.99. Prior to this analysis we were wondering whether the score improvements we were observing in Fig. 6 could also be due to the burial of exposed hydrophobic residues. Indeed, it is easy to understand why buried polar atoms are brought toward the surface by MD simulations. The energy potential includes explicit terms for electrostatics that favour the creation of hydrogen bonds or ionic interactions. On the other hand, no such explicit terms exist for the burial of non polar atoms. This burial is usually explained as the consequence of solvent entropic terms, i.e., the presence of non polar atoms in the solvent forces the water molecules to organise themselves around these atoms in a cage-like manner, which reduces their mobility and thus causes a decrease of the solvent entropy. To avoid this phenomenon, the solvent tends to expel non polar atoms from its bulk. It is thus very interesting to observe, as shown by the above analysis, that MD simulations in explicit solvent are able to reproduce this behaviour to a reasonable extent.

**Table 6:** Window score based on residue surface areas

	Sequence		E57	**F58**	**E59**	I60	**Y61**	**Y62**	I63	L64	**L65**	G66	**E67**
t = 0	Win. score	-2.61	-2.38	-2.15	-2.48	-2.36	-2.84	-2.74	-2.53	-2.45	-2.57	-2.50	
	Ind. score	-1.88	-3.06	-3.23	-1.36	-2.81	-4.72	-0.97	-2.34	-4.19	-0.69	-5.99	

t = 280	Win. score	-1.23	-1.18	-1.18	-1.26	-1.21	-1.28	-1.25	-1.21	-1.37	-1.44	-1.33	
	Ind. score	-0.95	-1.61	-0.67	-1.03	-1.38	-0.09	-0.93	-2.76	-2.52	-0.69	-1.37	

Query	Win. score	-1.47	-1.34	-1.36	-1.48	-1.48	-1.50	-1.46	-1.50	-1.00	-0.99	-1.20	
	Ind. score	-1.09	-1.10	-5.99	-0.82	-0.18	-1.18	-0.58	-1.07	-2.92	-0.69	-0.87	

Residue window centred at Tyr62. Ind score is the individual score for the residue; Win. score is the window score, i.e., the mean of the individual scores in a window of 11 residues centred at the residue of interest. The first 2 lines, labelled t = 0, correspond to the minimised initial conformation at t = 0 ps; the next 2 lines, labelled t = 280, correspond to the minimised conformation along the MD trajectory at 400 K that has the smallest value for the Tyr 62 window score (t = 280 ps snapshot); the last two lines, labelled query, correspond to the score values of the residues in their native structure for comparison purpose. Residues in bold are shown in Fig. 8

**Figure 9:**
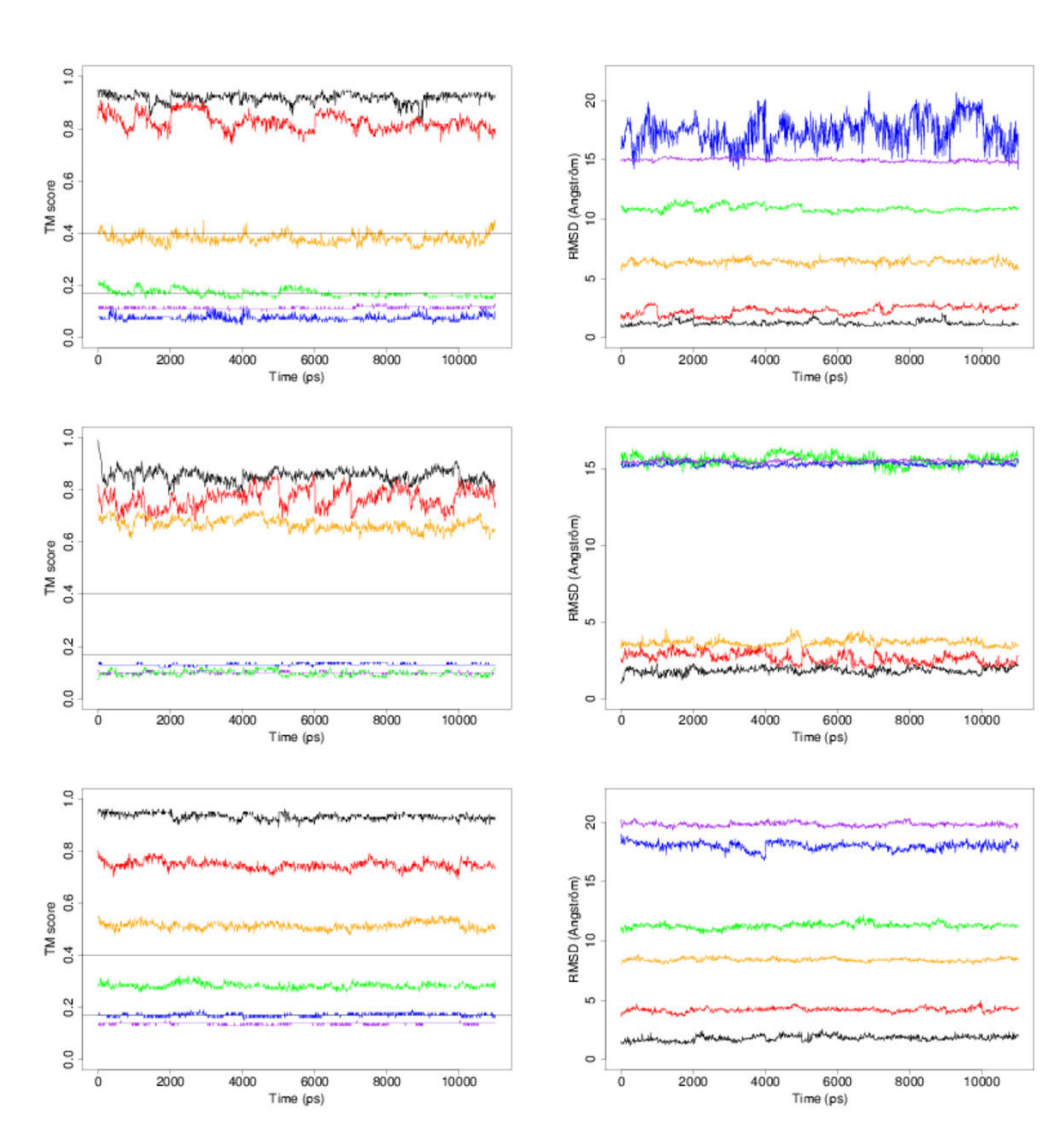
TM scores (left panel) and RMSd values (right panel) for the models of the all-*α *class (top), all-*β *class (middle) and *αβ *class (bottom). The colour code for the models is: NS0 = black, ST1 = red, ST2 = orange, ST3 = green, DT1 = blue, DT2 = purple. Horizontal lines in the TM score panel correspond to the limit above which two structures are clearly related in terms of 3D structures (0.4), and the limit below which they are clearly different (0.17).

Molecular dynamics simulations do improve the empirical scores, making the models more native-like, at least as far as residue surface areas are concerned. The question arises, therefore, of whether these simulations bring the models closer to the true native structure. Figure 9 presents the TM-scores and the RMSd values of the models of the three classes with respect to the corresponding *native structures*, along the trajectories. This figure shows that no such thing happens. It is not really surprising for DT and ST3 models that are very far form the true structure. For these models, getting closer to the true structure would imply large rearrangements of the polypeptide chain that are unlikely in 11 ns. However, for ST1 and ST2 models that are, presumably, in the basin of attraction of the native structure, MD simulations have no effect either. As shown in Fig. 8 above, the score improvement is usually due to localised rearrangements of the side-chains that do not affect much the polypeptide backbone conformation.

### Other criteria

We tried other criteria such as the fraction of side-chain contacts that are conserved along the trajectory or the radius of gyration of the molecules. These criteria proved to be not very efficient in discriminating between the different types of models (data not shown).

### Gibbs free energies

For the sake of comparison with published works we computed the Gibbs free energy (since our simulations were performed at constant pressure and temperature) for the different models along the trajectories at 300 K. It is important to notice that we only computed the free energy of the macromolecule. The Gibbs free energy is given by:

*G *= *U *- *TS *+ *PV*

where *U *is the internal energy, *T *is the temperature, *S *is the entropy, *P *is the pressure and *V *is the volume. In the calculation of the Gibbs free energy we omit terms whose contribution is the same for the different models. For instance, the internal energy includes the kinetic energy but since the temperature is fixed and models, for a given query sequence, have the same number of degrees of freedom the corresponding value is identical for all models. In a similar way, we assume that the PV term is very similar for all comparable models since there is no large change of volume involved, as this would be the case in a reaction producing gaseous compounds. We are, thus, only interested in terms that allow us to rank the different models of a given query sequence (to remind this we add a '*' to the Gibbs free energy: G*.

The internal energy therefore consists of 2 terms: the intramolecular energy and the energy of interaction of the molecule with the solvent. Both terms are computed as the corresponding mean energies of the conformations along the MD trajectory.

The entropy is computed as described in the Method section, taking advantage of the calculation and diagonalizing of the covariance matrix already performed.

For the reason invoked earlier, not all models corresponding to a given query sequence have the same number of residues. It is not possible to compare directly the energy or entropy terms of models having different numbers of atoms. Hereafter we only consider models with the same number of residues. Table 7 shows the results that were obtained.

**Table 7:** Gibbs free energies

			Energy of the protein			Energy protein-solvent					
Class	Model	Nres	*E*_0_	$\bar{E}$	*σ*_*E*_	*E*_0_	$\bar{E}$	*σ*_*E*_	TS	G*	$G_{0}^{*}$
all-*α*	NS0	105	-5 739	-5 316	345	-18 266	-17 123	672	1 157	-23 596	*-24 019*
	ST1	105	-4 002	-4 536	424	-20 587	-18 616	827	1 245	-24 397	*-23 863*
	ST2	91	-4 719	-4 892	335	-15 330	-15 354	636	1 119	-	*-*
	ST3	104	-3 041	-4 745	543	-21 442	-17 646	1 026	1 325	-23 716	*-22 012*
	DT1	105	-2 379	-4 627	400	-23 278	-17 894	798	1 483	-24 004	*-21 756*
	DT2	105	-5 018	-5 755	289	-16 742	-15 625	509	1 337	-22 717	*-21 980*

all-*β*	NS0	106	-6 018	-5 159	383	-15 237	-15 023	757	1 193	-21 375	*-22 448*
	ST1	106	-3 932	-4 836	333	-18 562	-15 585	657	1 274	-21 695	*-23 768*
	ST2	104	-2 828	-3 651	404	-18 910	-16 835	779	1 315	-21 801	*-23 053*
	DT1b	98	-3 068	-3 621	355	-17 127	-15 244	681	1 368	-	*-*
	DT1a	112	-3 301	-5 385	468	-19 417	-15 484	843	1 304	-	*-*
	DT2	106	-3 483	-4 426	374	-18 988	-16 233	704	1 185	-21 844	*-23 656*

*αβ*	NS0	260	-16 963	-13 912	452	-28 307	-30 284	826	2 339	-46 535	*-49 586*
	ST1	260	-11 223	-11 888	503	-36 671	-33 328	991	2 534	-47 750	*-47 085*
	ST2	260	-6 735	-9 817	911	-42 134	-36 954	1 625	2 731	-49 502	*-46 420*
	ST3	200	-6 774	-8 629	445	-28 844	-25 728	790	2 104	-	*-*
	DT1	196	-2 578	-5 515	907	-40 953	-34 756	1 653	2 382	-	*-*
	DT2	260	-8 369	-10 391	655	-40 331	-35 703	1 145	2 716	-48 810	*-46 788*

*E*_0 _is the energy for the initial conformation at t = 0; $\bar{E}$ is the mean energy along the trajectory at T = 300 K;*σ*_*E *_is the corresponding standard deviation. Column 3 is the number of residues in the model. Columns 4–6 correspond to the intramolecular protein energy, columns 7–9 correspond to the interaction energy of the protein with the solvent. Column 10 is the entropic contribution. Column 11, G* is the approximation of the Gibbs free energy (see text). Column 12, $\bar{E}$ is similar to G* except that it is computed using the protein is similar to *G *term *E*_0 _instead of $G_{0}^{*}$. The Gibbs free energy is not computed for models for which the number of residues differs by more than a couple of residues from the query sequence length. All figures are in kJ/mol.

For the intramolecular energy term, NS0 models are the only models to have an initial energy lower than the mean energy at 300 K. NS0 models are close to the native structure and thus they are supposed to have many favourable interactions. When we consider the energy of conformations along the trajectory we expect less favourable interactions to be generated due to the thermal fluctuations, thus leading to an energy value that is higher on average. However, other models show an opposite behaviour, the initial conformation has an energy higher than the mean energy. The initial conformation for these models is thus clearly not optimal in terms of the physics-based potential. NS0 models do not always have the lowest intramolecular energy, for instance the DT2 model for the all-*α *class and the DT1a model for the all-*β *class have a lower intramolecular energy.

On the other hand, for the solvent – protein interaction energy, all models, except the NS0 model of the *αβ *class, exhibit a lower energy for the initial conformation than for the mean energy along the trajectory, as expected. It must be noted that this term strongly favours the ST2 and DT models over the NS0 model for the *αβ *class. For other classes this trend exists but is less pronounced. Dominy and Brooks [12] using the CHARMM force field and a generalized Born implicit solvation term found that misfolded states were favoured by the solvation term. They attributed this fact to the mispairing of favourable intramolecular ionic contacts.

The entropic term is proportional to the number of conformational states available to the system at a given temperature. We thus expect NS0 models to exhibit a smaller value for this term than DT2 models. We observe a reasonable correlation between the entropic term values and the range of models (except for the all-*β *DT2 models which has the smallest entropic value).

We computed two values of the Gibbs free energy: G* forz which we use the mean intramolecular energy ($\bar{E}$) along the trajectory and $G_{0}^{*}$ for which we use the initial value of the intramolecular energy *E*_0_. Considering these two terms, we obtain very different pictures. For $G_{0}^{*}$, there is a perfect correlation between the model spectrum and the free energy values for the all-*α *class and a reasonable correlation for the two other classes (once again the DT2 models depart from the expected behaviour, they have a slightly lower free energy value than the corresponding ST2 models). On the contrary, for G* there is an anti-correlation for the all-*β *class, and to a lesser extent for the *αβ *class and no clear correlation for the all-*α *class. We note that G* provides a better approximation of the true free energy value than $G_{0}^{*}$ since the intramolecular energy, and thus the internal energy, *U*, is much better estimated by the mean intramolecular potential energy along the trajectory than by a single minimised conformation. The good correlation observed between $G_{0}^{*}$ and the spectrum of models for the three classes is accidental. It is essentially due to the fact that the initial conformation for the models, as described above, contains many constraints compared to the native structure. These constraints are relaxed during the MD simulation. Therefore the Gibbs free energy, G*, *computed for the macromolecule alone*, does not appear to be an efficient criterion to discriminate the different types of models. Since, in this calculation, we omit a very important term, the entropic contribution of the solvent, this is not very surprising (see Discussion below).

### Analysis of the DT2 models

The unexpected behaviour of DT2 models that were supposed to act as controls at one extremity of the model spectrum (the opposite extremity being the NS0 models) prompted us to analyse in detail these models for the all-*α *and all-*β *classes.

DT2 models are supposed to be worst than DT1 models because we just swapped the sequences without paying attention to the burial of charged residues or the exposure of non polar residues to the solvent. DT1 models, on the other hand, correspond to the first false positive in the ranked list of scores. The fold recognition algorithm provides the best score it can find and thus tends to avoid, as much as possible, such very unfavourable side-chain assignment. However, DT2 models unlike DT1 and ST models, were aligned with 100% of the selected template structure. We did not need to model loop conformations with Modeller. To analyse whether this point was crucial we built new DT2 models for the all-*α *and all-*β *classes with the same template structure except that, this time, the coverage was 86% (slightly above the lowest coverage used for models of these classes, 83% for the all-*α *ST2 model, see Additional file 1, Table S1). In practice we randomly chose 15 residues in the template structure loops and we left to Modeller the task of building the conformation of these residues. These new models were then submitted to MD simulations at different temperatures as previously described.

Table 8 presents the same data as in Table 2 for the new DT2 models. It shows that the new models behave more like the DT1 model than the ST models, as the initial DT2 models did previously. To better understand the reasons of these changes, we analysed the molecular dynamics trajectories for the initial and new DT2 models. The structural differences between these models are relatively slight.

**Table 8:** Root Mean Square deviations

	300 K		350 K		400 K	
	
New DT2 models	mean	sd	Mean	sd	Mean	sd
all-*α*	4.7	1.29	3.8	0.98	5.8	0.83
all-*β*	4.2	0.70	5.0	0.70	6.6	1.10

Same data as in Table 2 for new DT2 models.

**Figure 10:**
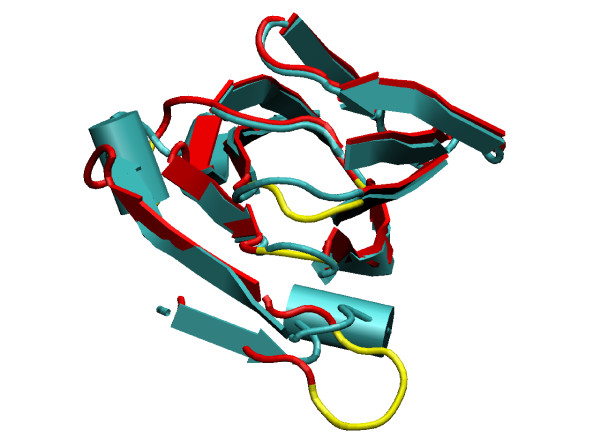
Superposition of the initial DT2 model for the all-*α *class, in cyan, with the new DT2 model, in red for the unmodified parts and yellow for the modelled loops. These 2 models differ in their coverage with respect to the template structure, 100% for the initial model and only 86% for the new model. Unaligned residues in the loops have been built with Modeller. They are shown in yellow. This figure was made with VMD [37].

For example, Fig. 10 shows the differences in terms of the 3D structure between the initial all-*α *DT2 model (in cyan) and the corresponding new model (in red for the aligned part and yellow for the modelled loops). The largest difference corresponds to the loop between the first 2 strands that is shown in the foreground of Fig. 10. In the template structure the loop is tight and there is a helix turn (represented by the cylinder in cyan) that packs against the rest of the structure. In the new model the loop conformation provided by Modeller is more extended and thus does not interact as closely with its counterpart in the template structure. When we monitor the trajectory for the new DT2 model we observe that the structure starts to unfold precisely at this point. The two first strands move apart from the other strands; they gradually unzip. This behaviour is not observed with the initial DT2 model, the helical turn remains firmly docked with the rest of the structure. A close inspection of the residues involved in the interface between these two portions of the 3D structure reveals that, just by chance, the interface consists of hydrophobic residues that are packed together.

For the all-*β *models the case is less striking. We let Modeller generate the conformation of 5 residues in each of the 3 inner loops, i.e., the loops between 2 helices. The conformation of the modelled loops appears to be more floppy than the one of the real loops. This is confirmed during the MD simulation since the modelled loops undergo large motions that lead, for one of the helices, to a tendency to unfold at both extremities (only the central part remains helical throughout the whole trajectory). Nevertheless, the helices, even though their local 3D structure is perturbed, remain docked together.

This experiment demonstrates that, although the fraction of the model backbone built by Modeller is low, these zones constitute weak points in the structure. It also shows that favourable interactions, even scattered in the 3D structure, can be sufficient to stabilise the model conformation, at least for a while.

## Discussion

In this work we performed MD simulations for different models and we monitored a number of structural properties along the corresponding trajectories to discriminate between correct and erroneous models. Before starting this work we had in mind the following picture about the probable behaviour of the different models during the MD simulations.

At one end of the model spectrum, for NS0 models, all side chains should pack nicely and, in general, most interactions should be favourable. Observing the energy hyper-surface at low resolution, this corresponds to a relatively deep minimum that we call hereafter the basin of attraction of the native structure. At higher resolution, this basin of attraction contains many local minima that are sampled at a given temperature. At 300 K we expect the system to remain within this basin during the whole simulation. In terms of structural properties this implies that the global organisation of the structure is left unmodified. This has for consequences that the RMSd fluctuations are relatively small, no large perturbation of the secondary structures is observed and very few residues are found in an unfavourable environment, as measured by their residue surface area. In addition, since only the portion of the conformational space corresponding to the basin of attraction of the native structure is available to the system, all the conformations that are sampled during the simulations should cluster in a limited number of groups. As described in the Result section, we can choose the clustering procedure such that only one group is obtained at 300 K.

At the other end, for DT models, since the sequences have been aligned with incorrect 3D structures, the packing of the side-chains should be far from optimal and a number of side-chains should be found in an unfavourable environment. We assume that the energy hyper-surface of such models is riddled with relatively shallow and broad minima that allow large fluctuations of the molecule. In the most favourable cases, we expect such models to hop from minimum to minimum and to gradually unfold during the simulations. Therefore we anticipate that the RMSd values with respect to the initial conformations will exhibit large drifts. A noticeable fraction of the initial secondary structures should be lost. Many residues should display low values of the empirical score based on the residue surface area. The number of conformation clusters should also be significantly larger, in agreement with the presence of many potential energy valleys easily reachable by the system.

Between these two extremes, other models should exhibit intermediate behaviours, depending on their proximity to NS0 or DT2 models.

We performed the simulations at several temperatures. We ignore the height of the energy barriers that separate the different valleys. In particular, large rearrangements of the polypeptide chain might correspond to relatively high energy barriers. Crossing such barriers is an event with a low probability of being observed in 11 ns. Raising the temperature amplifies the fluctuations. In this way, we hoped, for DT models, to be able to overcome energy barriers and start unfolding parts of the models.

Results presented above show that our view of the model behaviour was essentially correct.

As expected, the RMSd mean and standard deviation gradually increase from the NS0 models to the DT1 models (DT2 models act as outliers for the reasons we described above). However it is difficult to draw a clear line between the behaviour of DT and ST models. ST3 models tend to behave more like other ST models than DT models.

The number of clusters at 300 K would provide an excellent mean of discriminating ST from DT models but for the all-*β *DT1a and DT2 models that exhibit an anomalous behaviour. Raising the temperature at 350 K or 400 K solves this problem. This is one of the few cases where increasing the temperature is useful in these analyses. At 300 K ST1-2 models have less than 10 clusters, DT models several tens or hundreds. ST3 models are more difficult to classify, the all-*α *class ST3 model behaving like a DT model and the one of the *αβ *class rather like a ST model.

The usefulness of the secondary structure criterion to discriminate the models depends on the type of secondary structure. For models containing *α *helices there is a good correlation between the mean content in secondary structure and the spectrum of models. This is less clear for models containing *β *strands, in particular those of the all-*β *class. For this class the DT1a model and, once again, the DT2 model depart from the expected behaviour. Here also, performing the simulations at 350 or 400 K clarifies the situation. The surface area of hydrophobic/hydrophilic residues, implemented as a satisfaction score, provides the most quantitative criterion for the discrimination of the models. Using the threshold for the 1% confidence interval we are able to clearly distinguish ST1-2 models (except the ST2 model of the *αβ *class but as shown on Fig. 4 this model is far from the native structure with an RMSd of 8 Å) from DT models. With this criterion ST3 models behave like DT models.

To summarise the above points, no single criterion is sufficient to distinguish ST1-2 models from DT models in all cases. Criteria such as the RMSd mean, RMSd standard deviation and clustering are subject to a few false positives, i.e., DT models behave like ST models. The satisfaction score generates one false negative, e.g., the *αβ *ST2 model is indistinguishable from DT models with this criterion. The clustering and satisfaction score provide more quantitative criteria than other structural features we considered in this study. If we consider conjointly all the criteria it is relatively straightforward to discriminate ST1-2 from DT models, as shown in Table 9. Most of the time, ST3 models behave like DT models. Therefore grossly misaligning a sequence with the correct template structure results in a model that is no better than models for which the sequence has been aligned with erroneous structures.

**Table 9:** Criteria used to discriminate the models at 300 K

Class	Model	RMSd mean	RMSd fluct.	Clusters	SSEs	Satisfaction score
all-*α*	NS0	+	+	++	++	+++
	ST1	+	+	++	++	+++
	ST2	+	+	++	++	+++
	ST3	+	+	-	+	- -
	DT1	+	++	++	++	+++
	DT2	- -	-	++	++	+++

all-*β*	NS0	+	+	++	+	+++
	ST1	+	+	++	+	+++
	ST2	+	+	++	-	+++
	DT1b	-	-	- -	- -	+++
	DT1a	+	+	++	+	+++
	DT2	-	+	- -	- -	+++

*αβ*	NS0	+	+	++	++	+++
	ST1	+	+	++	++	+++
	ST2	+	+	+	+	- -
	ST3	+	+	+	+	- -
	DT1	+	+	++	-	+++
	DT2	- -	+	+	-	+++

RMSd mean, RMSd uctuations, number of clusters along the trajectory, evolution of the secondary structures, hydrophobic and polar satisfaction score. The number of '+' symbolises the confidence in the criterion result. For instance the hydrophobic/polar satisfaction score provides a quantitative answer that is easy to interpret whereas results provided by the RMSd mean are more difficult to judge. The '-' indicates false positives or negatives.

Running simulations at different temperatures did not prove very helpful, except possibly for the models of the all-*β *class for which, in a couple of occasions concerning the clustering and secondary structure criteria, it contributed to a better discrimination of the DT and ST models. Moreover, it is likely that 400 K is too high a temperature for running meaningful MD simulations.

Although the simulations were able to improve the surface area of hydrophobic/hydrophilic residues, making the side chains more native-like, we did not observe any improvement of the ST1-2 model backbones in terms of RMSd values, that is, the model main chains did not become any closer to the native structure main chains. MD simulations in explicit solvent using Rosetta decoys as models [15] showed that such improvements can sometimes be observed on a 10–100 ns time scale. In this study the simulation time corresponds to the lower end of this interval which might explain, in part, why we do not witness any backbone improvement. Other works, using Monte Carlo simulations [24] or just simple minimisations [25], did succeed in improving the ranking of good models and, sometimes, in refining the conformation of "low" resolution 3D structures [24,26]. In our case our "good" models are relatively far from the native structures (the ST2 model for the all-*α *class is 5.7 Å and the ST2 model for the *αβ *class is 8 Å from the corresponding native structures). It is likely that this sort of "low" resolution models need more than 11 ns to be significantly ameliorated.

Computations of the Gibbs free energy did not help us in discriminating correct from erroneous models. Other studies arrived at a different conclusion, based on the analysis of a *single *minimized conformation using molecular mechanics energy functions complemented with different ways of computing solvation terms.

As observed in Table 7, there is a large, favourable, intramolecular energy gap between the initial conformation of the NS0 models and all other models for the three classes. This is, no doubt, due to the fact that, even though the initial conformations have been minimized using the physics-based energy potential, there remains many constraints in the models that were not built from the native structure. This is the reason why, as described in the Result section, the mean intramolecular energy for NS0 models is higher than the initial conformation energy whereas the opposite is true for all other models. However, during the MD simulations, the systems have enough time to relax and to improve their conformations. As a result, the gap between the mean intramolecular energies for the NS0 models and other models narrows, blurring the different between the different models. This point is clearly highlighted by our computation of the free energy ($G_{0}^{*}$) using the initial intramolecular potential energy, E_0_, instead of the more correct mean intramolecular potential energy, $\bar{E}$, along the MD trajectory. Considering $G_{0}^{*}$ instead of G* we have quite a good correlation, albeit fortuitous, between the free energy values and the model spectra.

In addition, the protein-solvent energy shows a tendency to favour the misfolded states. This fact has already been pointed out by Dominy and Brooks [12]. In this study, this is particularly pronounced for models of the *αβ *class. The contribution of this energy term to the Gibbs free energy is important since, on average, it is about three times larger than the corresponding intramolecular energy term.

As expected, the entropic energy term favours, to a large extent, DT models but the difference between these models and NS0 models is small, since all models correspond to folded, compact, conformations. Therefore this leads us to the apparently paradoxical situation where the more correctly computed Gibbs free energy G* appears to be anti-correlated with the range of models for the all-*β *and *αβ *classes, and not correlated for the all-*α *class. This problem is mostly due to the protein-solvent interaction.

In fact we know that the whole system, protein *plus *solvent, should be taken into account in the free energy calculations. In particular, as described in the Result section, it appears crucial to include the solvent entropic contribution to obtain consistent Gibbs free energy values. However computing this term, when an explicit solvent is employed, is difficult since we cannot use the methodology we adopted for the protein (i.e., computing the vibrational entropy in a quasi-harmonic approximation of the molecular motion). Using an implicit solvent model and computing the corresponding solvation free energy might be a solution. However, we would like to draw attention to the fact that, in order to compute the intramolecular energy of the protein one should not consider only the initial conformation obtained with a modelling program but should use the mean of this energy along the MD trajectory.

## Conclusion

The principal question we wanted to answer with this work was: can we discriminate correct from erroneous models by monitoring the behaviour of structural properties along the MD trajectories? This constitutes more than a rhetorical question for us since we wish to apply this technique to a number of models built from the alignments we obtain with our fold recognition method, FROST. For some of these alignments whose score lies in the FROST twilight zone we cannot be sure whether we have aligned the sequence with the correct template structure or not. Results obtained in this study show that, although no single criterion is 100% efficient in this task, considering them in combination, as shown in Table 9, allows us to readily discriminate the two types of models. Another question we had, concerning the possibility of detecting zones of the model corresponding to local misalignments of the sequence onto a correct template, mostly received a negative answer. When the alignment contains severe errors the resulting model cannot be distinguished from plain erroneous models. We were surprised to observe that the difference of behaviour during the simulations between the ST and DT models was more gradual than we expected. Before undertaking this study we thought that it would be easier to witness the unfolding of DT models on a 10 ns time scale than it proved to be.

We must stress that, although we did our best to analyse several types of proteins with various secondary structure contents, e.g., all-*α*, all-*β *and *αβ *domains, the conclusions we draw from this study are based on a limited set of examples. Further work is needed to confirm the trends we identified here. Another important point to underline is the fact that these conclusions are dependent on the modelling program and, to a lesser extent, the MD simulation protocol used. We employed Modeller to build the models. This program provides good models with reasonable native-like structural features except, maybe, for some of the loop conformations. Using another, less efficient, modelling program might have changed the conclusions. This fact must be kept in mind when one is comparing different studies about the stability, or the energy, of models.

Our aim, when performing these simulations on models, is not so much to verify that the models are reasonable in terms of structural properties as to check whether our fold recognition method has been able to identify correctly the template structure. We are interested in microbial genome annotation [27]. Homology search methods constitute the cornerstone of the annotation process. Unfortunately, methods based on sequence comparison are no longer efficient when the similarity is below 20% sequence identity, leaving between 25 to 50% "orphan" coding sequences (CDSs), depending on the organism analysed. Fold recognition techniques can detect remote homologs, having less than 20% sequence identity, allowing us to obtain a crucial piece of information about these orphan CDSs. Therefore the MD simulations we described in this study are a means for us to improve the sensitivity and specificity of our fold recognition method for ambiguous cases, i.e., when the corresponding scores are within the twilight zone.

To conclude with a practical consideration, we give an order of magnitude of the amount of time required for testing one model. For an all-*α *model, the system, protein plus solvent, is made of about 15,000 atoms. We have available a cluster of 10 bi-processors dual core x86 64 at 2.33 GHz (10 blades). Using the 4 CPUs of a blade in parallel we are able to simulate 8 ns a day (2.6 ns a day per CPU). Although not exactly a routine calculation, this allows us, to run an 11 ns MD simulation for a model of this size, overnight, employing 3 blades. The analysis of the trajectories, due to the minimisation of the conformations, requires about the same amount of time. A way of speeding up the simulations would be to use an implicit representation of the solvent instead of the explicit representation we used in this study. This would have the additional merit of allowing us to compute the solvation free energy.

## Methods

### Selection of query and template structures

For the selection of the query domains we started with two folds belonging to three SCOP classes : DNA/RNA binding 3 helical bundle (81 families) and 4 helical up and down bundle (12 families) for the *α *class; immunoglobulin-like beta sandwich (49 families) and double stranded *β *helix (29 families) for the *β *class; TIM barrel (84 families) and NADP binding Rossmann (29 families) for the *αβ *class.

Representatives of the different families of these folds were selected with the help of the AEROSPACIE score computed for the ASTRAL database [28]. As far as possible, we disregarded domains with prosthetic groups, disulfide bridges, co-factors and those belonging to oligomers.

A cross-comparison of all these representatives was carried out using the 3D structure comparison method VAST [29,30] to measure their degree of structural relatedness and to obtain 3D structural alignments. To complement the results provided by VAST we also employed another structural measure: the TM-score [31].

We then used the fold recognition program FROST [17] to obtain, for each representative domain, a list of template structures ranked by score. Representative domains were tagged as potential queries if we could find, in the top of the corresponding lists, three structures similar to the representative domain fold and with the following properties. The first similar structure (called Similar Template 1, ST1) had to be close to the representative domain with a high percentage of identical residues (for fold recognition techniques this corresponds to a range of 20–30% sequence identity) and an alignment close to the structural alignment found by VAST between the pair of structures. The second similar structure (ST2) had the same characteristics as ST1 except for the percentage of sequence identity that had to be low (typically less than 20% identity). Finally for the third similar structure (ST3) the alignment provided by FROST had to contain errors with respect to the structural alignment found by VAST.

For each of the three query structures selected in such a way (one for each class of secondary structure: *α*, *β*, *αβ*) we built six models (as described in the next section). The first one corresponded to the native structure (called NS0), the next three to the similar structures (ST1, ST2, ST3). The fifth one corresponded to the first false positive in the list of template (see Fig. 1), i.e., a structure having no relation with the query structure (called Different Template 1, DT1). The last one (called DT2), used as a control, was similar to the experiment of Novotny *et al.*[5]: we just aligned, without insertion/deletion, the query domain sequence with a fold having a similar size and belonging to a different secondary structure class. Table 1 summarises the characteristics of the different models that were built.

### 3D structure modelling

Three-dimensional models were built with Modeller 6v1 [32] starting from the alignments provided by the fold recognition method FROST (except for DT2 models and the ST1 models as discussed in the Result section). For each type of model, five models were generated using Modeller default parameters and the one having the best objective function was kept. For the query sequence we did not use the PDB structure directly. We created a 3D model using the same modelling protocol starting from a (perfect) alignment of the sequence with itself. For DT2 models we aligned the query sequence with the sequence of the template structure without insertion/deletion since both sequences have approximately the same number of residues. For ST1 models, to be sure to obtain good alignments, we used the more accurate VAST structural alignment instead of the FROST alignment.

### Molecular dynamics simulations

All simulations were performed in explicit water using the version 3.3.1 of the Gromacs package [33] in conjunction with the Gromos96 43a1 force field for condensed phase. The Simple Point Charge (SPC) model was used to represent water. The protonation state of ionizable groups in the proteins was chosen appropriate for pH 7.0. Counterions were added to neutralise the system. Molecular dynamics simulations were performed at constant temperature and pressure in a periodic rhombic dodecahedron box. The minimum distance between an atom of the protein and a box wall was 0.8 nm. Simulations at 300, 350 and 400 K were carried out. During these simulations a pressure of 1 bar was maintained. Temperature and pressure were kept constant by coupling to an external heat bath (Berensen thermostat) and an isotropic pressure bath (Berensen barostat). Corresponding relaxation times were set to 0.5 ps (separately for the protein, the solvent and the counterions) and 1.0 ps. Electrostatic interactions were calculated with the fast Particle-Mesh Ewald method (PME) and a distance cut-off of 0.9 nm. When using PME, other relevant parameters such as FFT grid spacing, interpolation order, geometry of the Ewald summations, etc. were left to their default values. Van der Waals (VdW) interactions were evaluated using twin range cut-offs of 1.4 nm for VdW cut-off and 0.9 nm for the short-range neighbour list. Non-bonded interactions were evaluated with a grid using periodic boundary conditions in all directions. Neighbour pair list was updated every 10 steps. Covalent bonds with H-atoms were constrained using the LINCS algorithm. An integration step of 2 fs was used. To generate the starting conformation the following protocol was employed. First an energy minimisation was performed using steepest descent followed by a quasi-newton method (Broyden-Fletcher-Goldfarb-Shanno algorithm). This phase was followed by 200 ps of equilibration with position restraints of the protein using a simulated annealing procedure that, starting at a temperature of 250 K, raised it to 300 K in 10 ps and maintained it to this value for the 190 remaining ps, allowing the system to relax. Production phase data were obtained by first carrying out a 1 ns simulation run then performing ten 1 ns simulation runs branching off this first trajectory every 100 ps. This procedure was used to improve the sampling of the conformational space [34]. The total length of the production phase trajectory was thus 11 ns.

### Principal component analysis

Principal component analysis, or covariance analysis, consists in calculating the mass-weighted covariance matrix of the atomic coordinates along the MD trajectory then diagonalising it. The eigenvectors are the essential modes that describe collective motions in the protein. The corresponding eigenvalues are the mean square fluctuations along these modes. The main advantage of this technique is that only a very limited number of modes (at most a dozen) are required to describe most of the protein dynamics. Using these data different analyses can be carried out: projection of the trajectory along the different modes, atomic mean square fluctuations along the modes, subspace overlap, etc. A very useful analysis is the calculation of the cosine content of the first principal components that gives an indication of the efficiency of the conformational space sampling. To perform the analysis described in the text we used the relevant Gromacs tools, in particular g_covar to calculate and diagonalise the covariance matrix and g_anaeig to analyses eigenvectors and eigenvalues.

### Computation of the vibrational entropy

The mass-weighted covariance matrix can be used to compute the vibrational entropy of the molecule. The molecular motion along the MD trajectory is approximated as quasi-harmonic. Covariance matrix eigenvalues, *λ*_*i*_, define the corresponding frequencies:

$${\left(2\pi \nu_{i}\right)}^{2}=\frac{k_{B}T}{\lambda_{i}}$$

the entropy *S *of the macroscopic state is given by [35]:

$$\begin{array}{ccc} S=N_{A}k_{B}\sum_{i}\frac{\theta_{i}}{\exp(\theta_{i})-1}-\log(1-\exp(-\theta_{i})) & \text{where} & \theta_{i}=\frac{h\nu_{i}}{k_{B}T} \end{array}$$

*k*_*B *_is the Boltzmann's constant, *h *the Planck's constant, T the temperature and *N*_*A *_the Avogadro's number.

### Clustering of conformations

Conformations every 10 ps along the trajectories were minimised in vacuum. The resulting minimum conformations were clustered using the Gromacs tool g cluster that provides several clustering methods. Here we build clusters with a single linkage method using, for each class, a cut-off value corresponding to the RMSd mean value at 300 K for NS0 models: 1.2 Å, 1.86 Å and 1.98 Å for the all-*α*, all-*β *and *αβ *classes respectively. The RMSd mean values used are those obtained with the minimised conformations. In a real situation, since we do not know the native structure of the query sequence, we would use RMSd cut-off values of 1.5 Å for all-*α *proteins and 2.0 Å for all-*β *and *αβ *proteins.

### Statistical score based on the residue surface area

To analyse the quality of models we developed an all-atom statistical score based on the residue surface area (unpublished work), inspired by the work of Eisenberg and colleagues in [36]. Here, for completeness, we give a brief description of this statistical score.

For each type of residue we compute the fraction of the polar atom surface in contact with other polar atoms, dubbed "polar satisfaction" and the fraction of non polar atom surface in contact with non polar atoms, called "hydrophobic satisfaction". We consider oxygen and nitrogen as polar and all other atoms as non polar. Hydrogen atoms are ignored in this analysis. These two terms measure the tendency of non polar atoms to form a hydrophobic core and of polar atoms to avoid being buried among non polar atoms. We compiled these fractions for residues belonging to three sets of proteins (all-*α*, all-*β *and *αβ*) as shown in Table S4 in Additional file 1. The mean environment for residues in a protein depends on the size of the protein thus we only selected proteins whose length was ± 30 residue long with respect to the length of the three query proteins. The proteins are surrounded by water molecules, i.e., polar atoms.

In Additional file 1, Fig. S8 shows the calculated density and cumulative probabilities for the tryptophan hydrophobic satisfaction in proteins belonging to the all-*α *class. Let us assume that, for a tryptophan in a model, we measure a hydrophobic satisfaction value of 0.706 as shown on Fig. 8. The corresponding score is the logarithm of the cumulative probability for the hydrophobic satisfaction (HS) up to the measured value, i.e., *S *= *ln*(*p*(*HS *< 0.706)) = *ln*(0.57) = -0.56. With this definition, if a hydrophobic residue is completely buried among hydrophobic atoms, its hydrophobic satisfaction is 1 and hence its score is maximum (zero). The lower the hydrophobic satisfaction is the lower the score. The same score definition, only based on the polar satisfaction, apply for polar residues. In our analysis we considered {A, V, C, I, L, F, M, Y, W, P} as hydrophobic residues and {S, T, H, K, D, N, Q, E, R} as polar residues. Notice that polar residues consist of polar and non polar atoms. In this analysis we did not take into consideration non polar atoms of polar residues. Tryptophan and tyrosine, in spite of having one polar atom, are considered as hydrophobic due to the large number of non polar atoms that compose their side chain.

For proteins of the three sets we computed the distributions of scores for the whole protein (global score) as well as the distributions of scores for windows of width 11 residues along the sequence. The latter is intended to pinpoint zones in the model for which residues significantly depart from the behaviour observed in protein 3D structures. In Additional file 1, Table S3 shows the mean, standard deviation and the value corresponding to 1% confidence threshold for the global score and window score. The Shapiro-Wilk statistical test computes the p-value that the distribution is normal (null hypothesis). For the all-*α *and *αβ *protein global score distributions one cannot reject the null hypothesis at a confidence threshold of 10%. This is not true for the all-*β *proteins. For them we computed empirically the 1% confidence interval. For the window score distributions, there is a strong correlation between overlapping windows and it is likely that the Shapiro-Wilk test is meaningless in such a case. We also computed empirically the corresponding 1% confidence level. It is interesting to notice that the means are all very similar for the global or the window scores. The window score distributions have all the same standard deviations. For the global score distributions the standard deviations decrease from the all-*α *class to the *αβ *class. This is due to the number of proteins involved in the computation that goes from 79 to 167 and also to the size of the proteins that varies from 106 to 230 residues. In Additional file 1, Table S3 indicates that residues, on average, have similar environments whatever the structural class considered.

## Authors' contributions

JFT participated in the conception of the study, carried out the analyses and participated in the writing of the manuscript. AM provided assistance with the FROST program. JFG conceived of the study, supervised and coordinated the whole project and wrote the manuscript. All authors read and approved the manuscript.

## Supplementary Material

Additional file 1 Supplementary tables and figures. This file contains the 8 supplementary figures and 4 supplementary tables listed in the text. Click here for file
