# Supplementary material for: Can molecular dynamics simulations help in discriminating correct from erroneous protein 3D models?
Source: BMC Bioinformatics. 2008 Jan 7;9:6. doi: 10.1186/1471-2105-9-6 (PMC2245900; doi:10.1186/1471-2105-9-6)
Supplement: Additional file 1 — Supplementary tables and figures. This file contains the 8 supplementary figures and 4 supplementary tables listed in the text. [file 1471-2105-9-6-S1.pdf]

1 Supplementary tables

Table S1: **Structural similarity between the queries and the templates according to FROST and VAST**

Class	Model	SCOP domain	FROST %Id	FROST S	FROST %Cov	FROST TM	FROST RMSd	VAST %Id	VAST S	VAST %Cov	VAST TM	VAST RMSd
all-α	NS0	1tqgA	100	93.9	100	1.00	0.0	100	4.5	100	1.00	0.0
	ST1	1i5nA	32	75.3	99	0.87	1.8	33	3.6	95	0.89	1.4
	ST2	1ls1A1	5	8.9	83	0.41	5.9	4	-0.4	45	0.62	2.7
	ST3	1jr8A	4	8.9	93	0.22	9.6	13	-0.2	36	0.43	2.5
	DT1	1s7oA	10	7.8	98	0.07	17.0	13	-0.7	29	0.49	2.1
	DT2	1o4tA	-	-	-	-	-	-	-	-	-	-
all-β	NS0	1o4tA	100	77.1	100	1.00	0.0	100	13.0	100	1.00	0.0
	ST1/ST2	1vj2A	18	16.8	96	0.71	3.4	22	10.0	77	0.90	1.4
	DT1a	1tzaA	10	12.9	88	0.08	16.0	4	0.3	34	0.35	3.9
	DT1b	1mjuL1	6	8.3	97	0.13	15.0	3	1.6	29	0.42	4.0
	DT2	1tqgA	-	-	-	-	-	-	-	-	-	-
$\alpha\beta$	NS0	1k77A	100	194.0	100	1.00	0.0	100	23.0	100	1.00	0.0
	ST1	1i60A	16	8.9	98	0.69	6.8	18	16.0	95	0.83	3.0
	ST2	1qtwA	6	9.8	96	0.54	8.0	12	13.0	91	0.79	3.3
	ST3	1qpoA1	9	9.3	63	0.29	11.0	6	8.0	49	0.67	3.5
	DT1	1m4oA	7	8.3	60	0.17	18.8	5	-4.8	7	0.04	3.8
	DT2	1bkpA	-	-	-	-	-	-	-	-	-	-

%Id: Percentage of sequence identity; S: Method score; %Cov: percentage of the query sequence aligned with the template; TM: TM-score between aligned segments; RMSd: Root Mean Square deviation corresponding to aligned residues (in Å).

Table S2: **Comparisons of the models and the query native structures**

Class	Model	TM	RMSd	Nres	$N\alpha$	$N\beta$	Ncoil
all-α	NS0	0.99	0.5	105	90	0	15
	ST1	0.86	1.8	105	85	0	20
	ST2	0.42	5.7	91	65	0	26
	ST3	0.24	11.1	104	76	0	28
	DT1	0.08	17.0	105	78	0	27
	DT2	0.11	15.0	105	6	66	33
all-β	NS0	1.00	0.2	106	3	66	37
	ST1	0.83	2.3	106	0	56	50
	ST2	0.71	3.4	104	0	59	45
	DT1a	0.08	16.0	98	0	59	39
	DT1b	0.13	15.0	112	0	48	64
	DT2	0.11	15.3	106	86	0	20
$\alpha\beta$	NS0	1.00	0.1	260	110	36	114
	ST1	0.81	3.4	260	114	44	102
	ST2	0.54	8.0	260	107	29	124
	ST3	0.29	11.1	200	62	26	112
	DT1	0.17	18.8	196	30	14	162
	DT2	0.13	20.6	260	84	48	128

TM: TM-score; RMSd: Root Mean Square deviation in Å; Nres: Number of residues in the model; $N\alpha$, $N\beta$, Ncoil: number of residues in the corresponding secondary structures.

Table S3: **Score distribution characteristics**

Score	mean	std. dev.	1% conf.	N	Shapiro
$S_{prot}\alpha$	-0.99	0.130	-1.29	79	0.8
$S_{w11}\alpha$	-0.98	0.296	-1.87	7297	—
$S_{prot}\beta$	-0.98	0.113	-1.30	118	< 0.01
$S_{w11}\beta$	-0.96	0.293	-1.81	11 779	—
$S_{prot}\alpha\beta$	-0.98	0.076	-1.15	167	0.1
$S_{w11}\alpha\beta$	-0.97	0.301	-1.83	42 198	—

S_{prot} global score, S_{w11} window score, mean: average value, std. dev.: standard deviation, 1% conf: 1% confidence value, N: number of points involved in the computation, Shapiro: Shapiro-Wilk p-value, when applicable.

Table S4: **Proteins used for the analysis**

class	Nprot	Nres	Lp min	Lp max	% seq ID
all-α	79	8 087	70	130	50
all-β	118	12 959	70	130	50
$\alpha\beta$	167	43868	230	290	50

Nprot: number of proteins used in the analysis, Nres: corresponding number of residues, Lp min: minimum size (in residues) of the proteins, Lp max: maximum size of the proteins, % seq ID: maximum percentage of identity between 2 proteins.

2 Supplementary figures

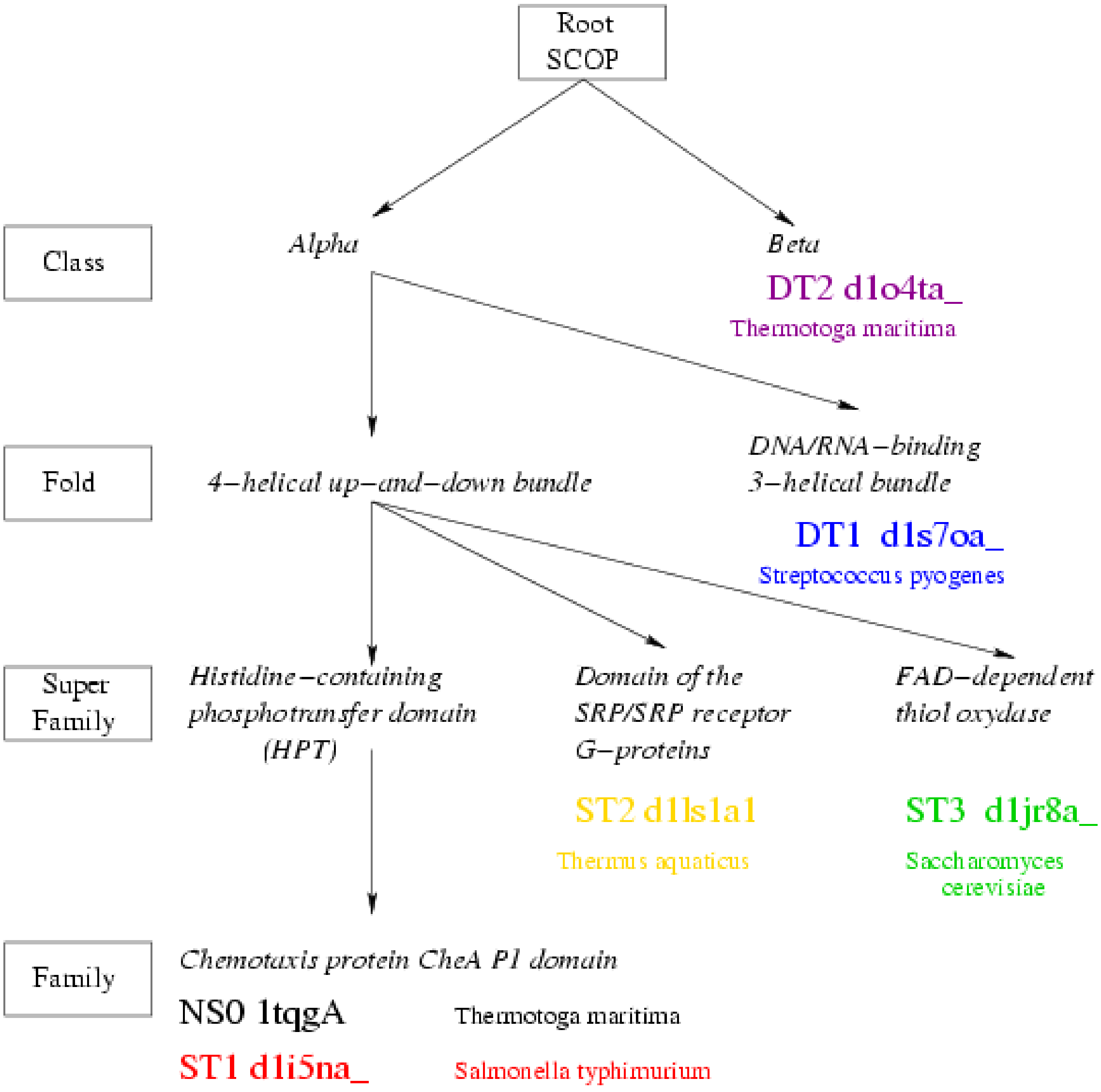

Figure S1: Relationships between the query and the template structures for the all-α class according to the SCOP hierarchy.

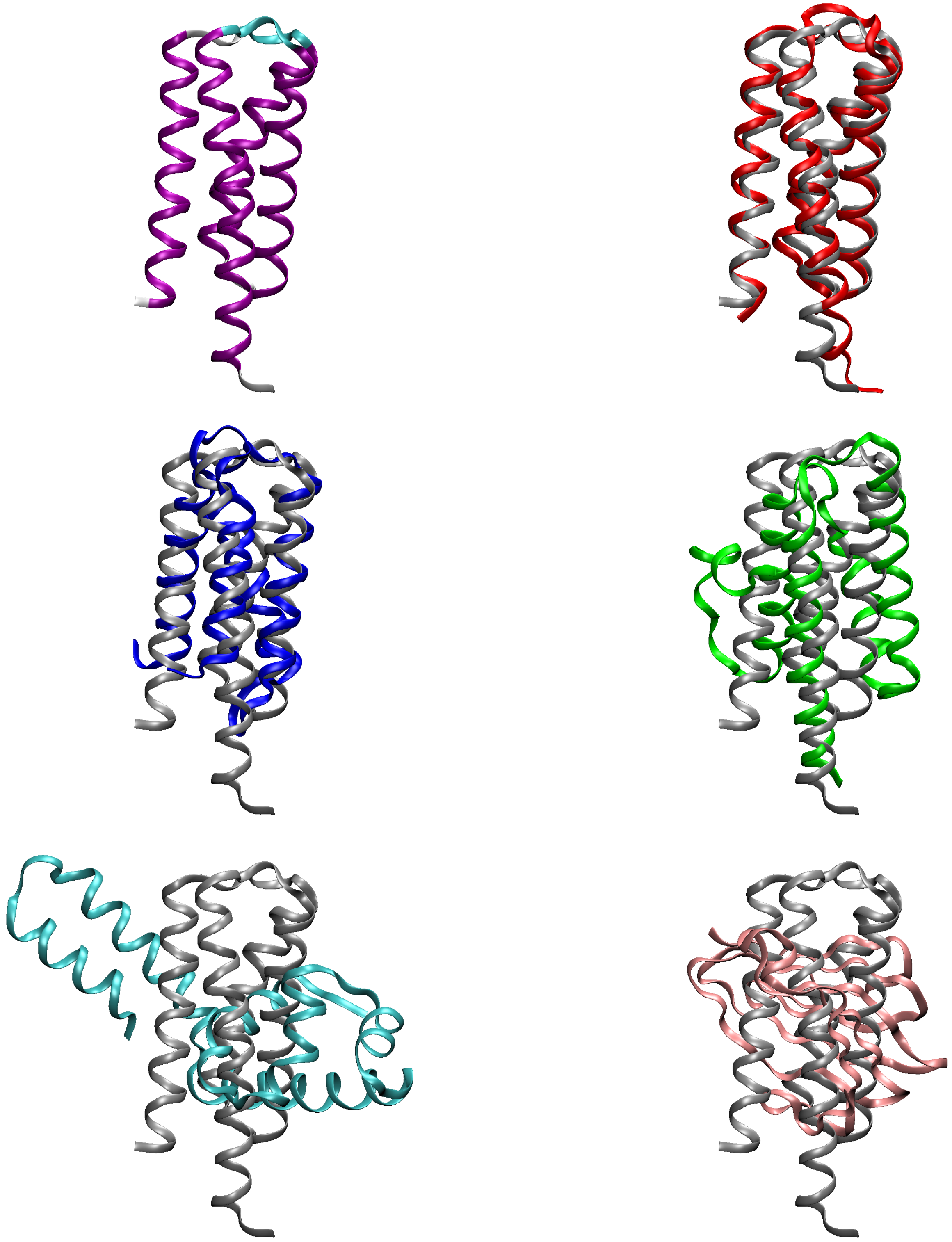

Figure S2: Superposition of the 6 models with the query native structure for the all-α class. From top to bottom and left to right: NS0, ST1, ST2, ST3, DT1, DT2

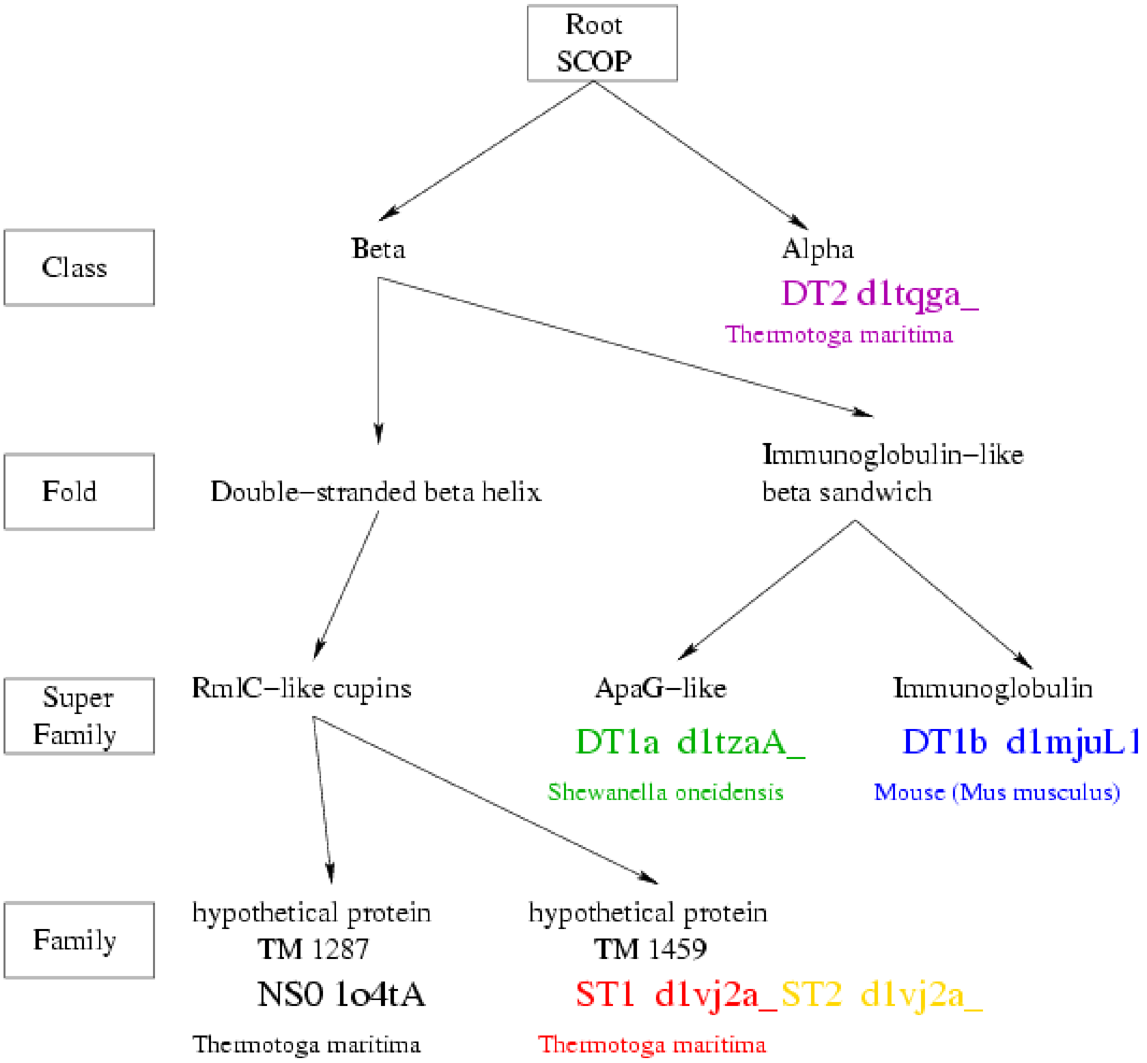

Figure S3: Relationships between the query and the template structures for the all-β class according to the SCOP hierarchy.

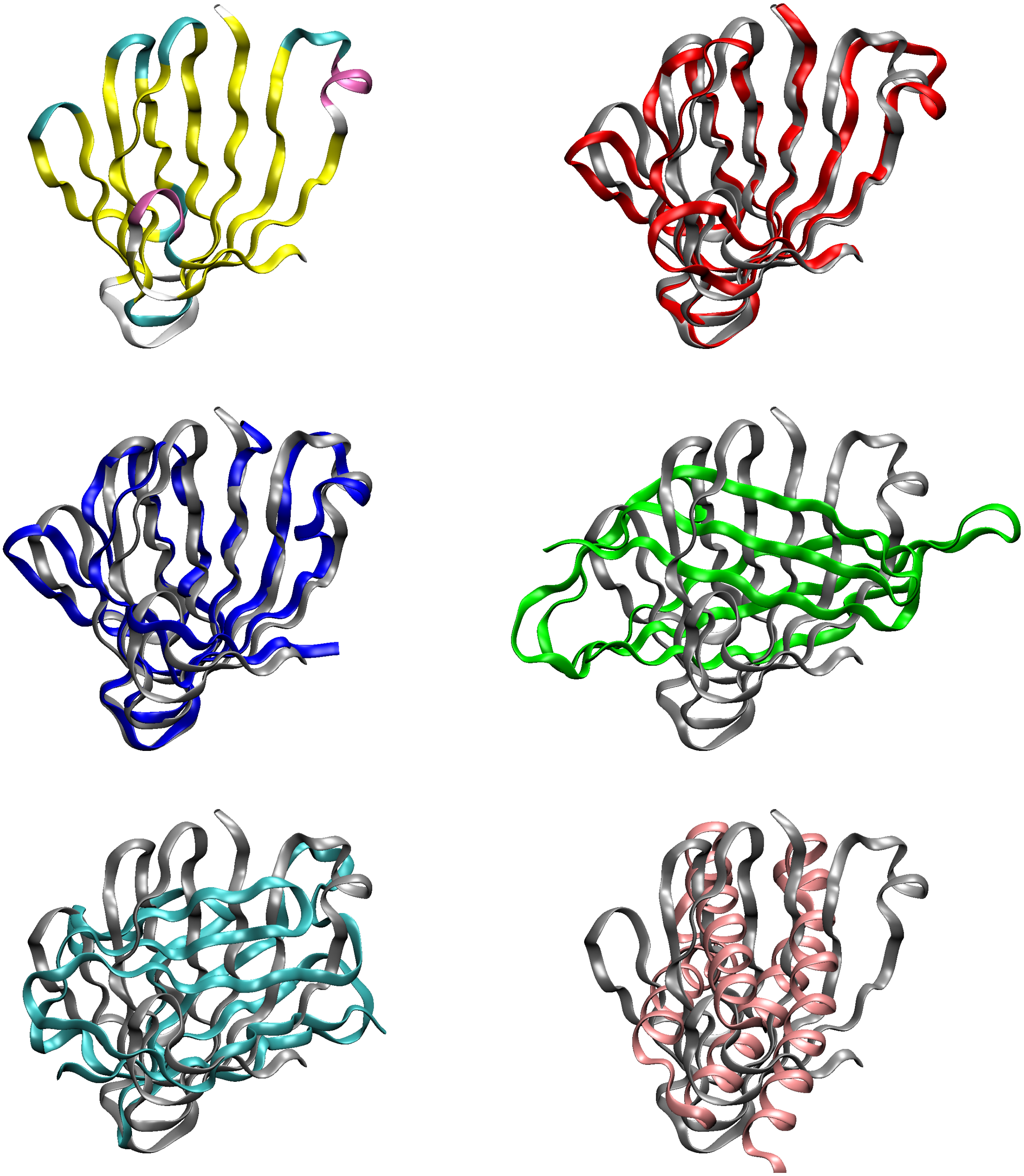

Figure S4: Superposition of the 6 models with the query native structure for the all-β class. From top to bottom and left to right: NS0, ST1, ST2, DT1a, DT1b, DT2

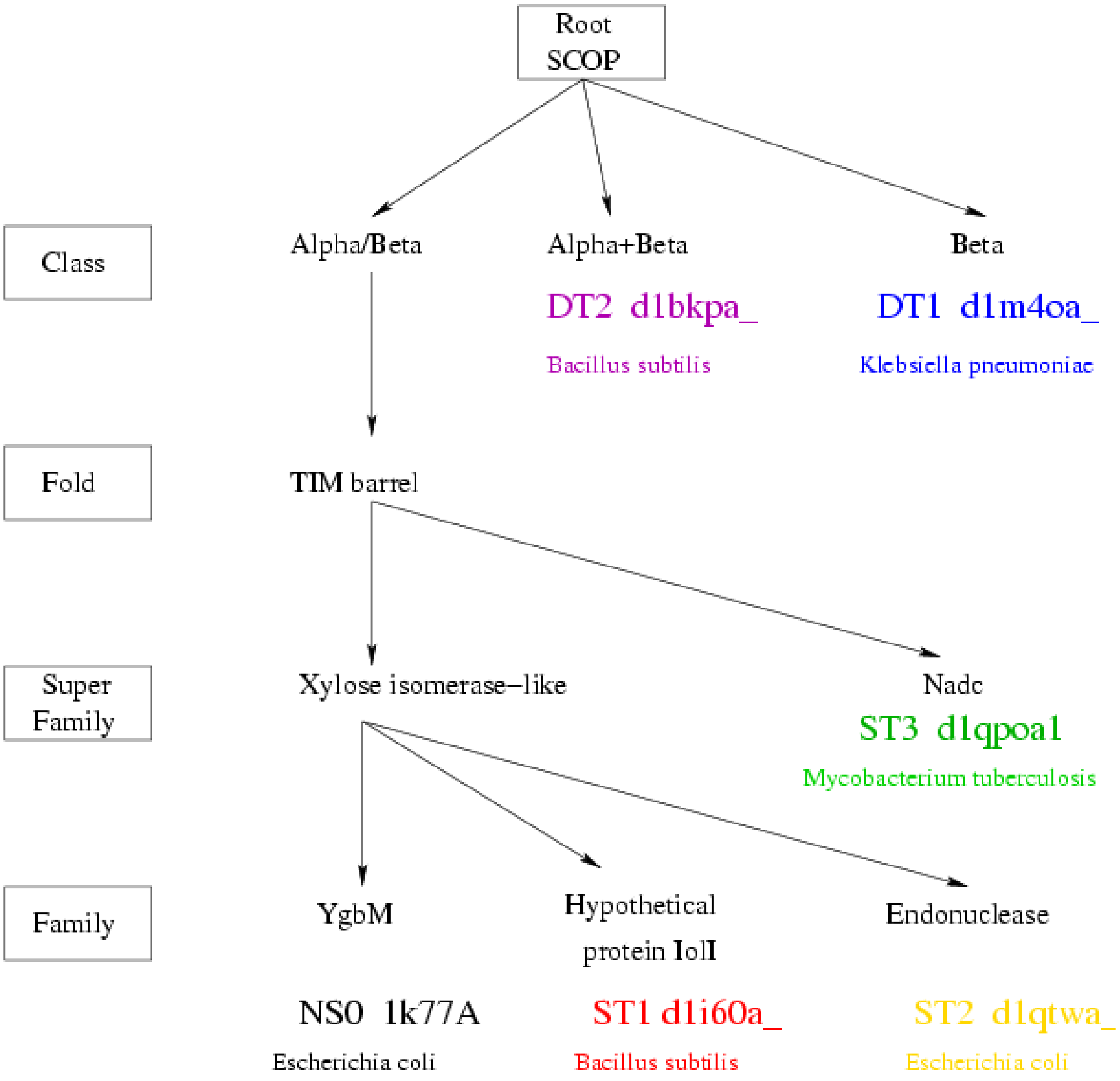

Figure S5: Relationships between the query and the template structures for the $\alpha\beta$ class according to the SCOP hierarchy.

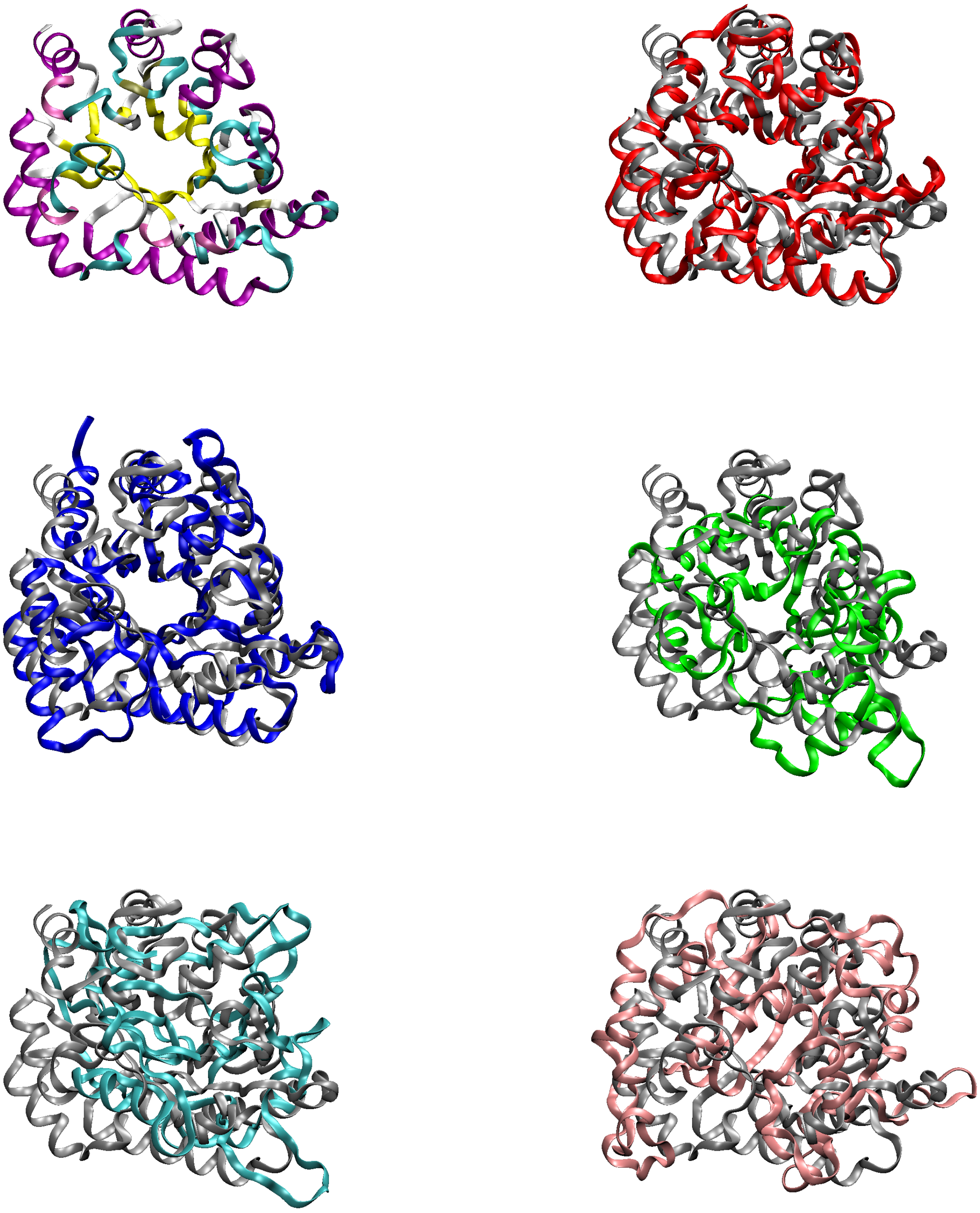

Figure S6: Superposition of the 6 models with the query native structure for the $\alpha\beta$ class. From top to bottom and left to right: NS0, ST1, ST2, ST3, DT1, DT2

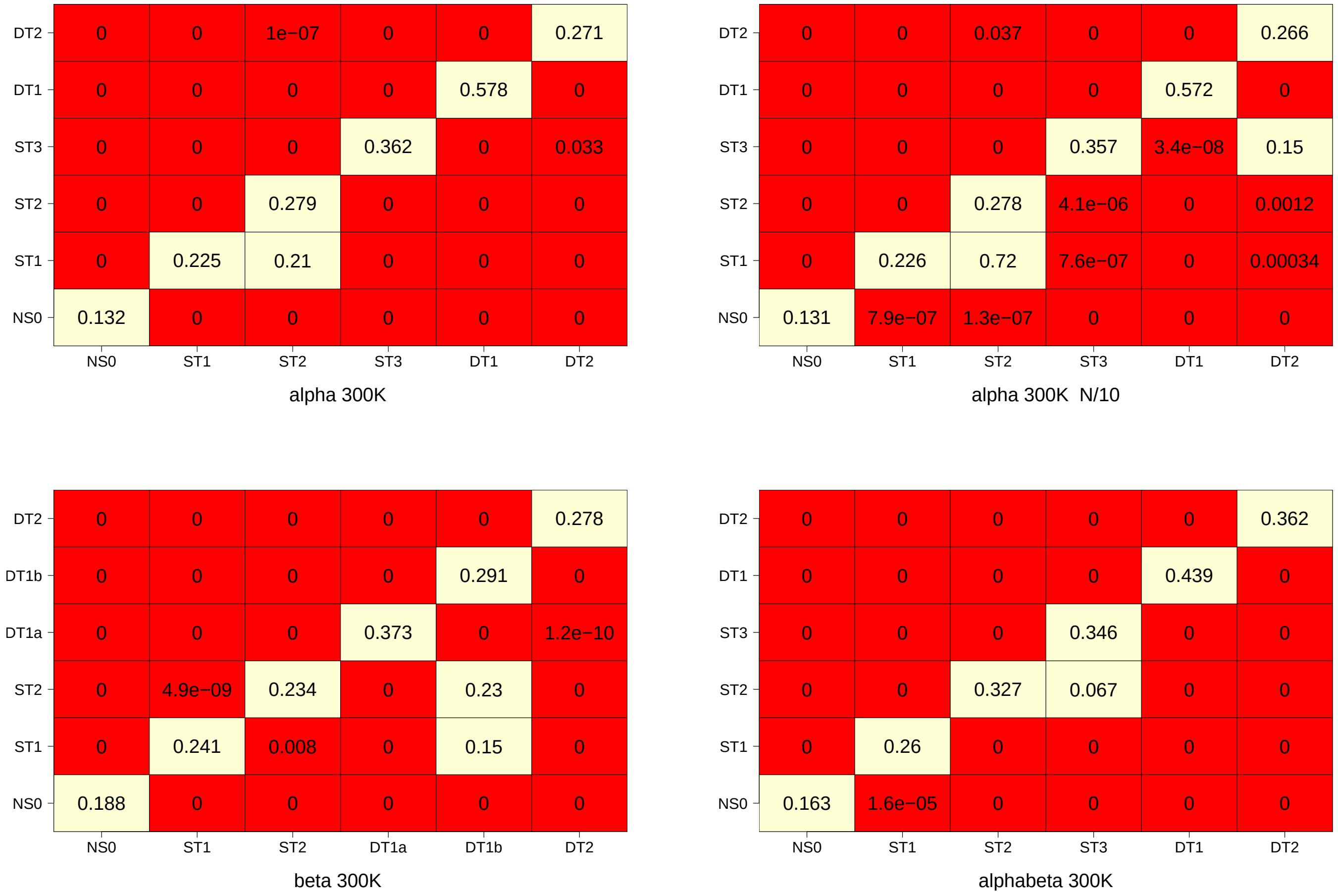

Figure S7: Student p-value matrices for the all-α class (top left), all-β class (bottom left) and $\alpha\beta$ class (bottom right) at 300 K. The top right matrix is similar to the top left matrix except that the number of points used for the computations is ten times less. The matrix upper triangular values show the P-values for the Student t-test on the means. The lower triangular values shows the p-values for the Student F-test on the variances. Diagonal elements show the RMSd mean values in nm. For instance the first row, third column position of the top left matrix whose value is $1\,10^{-7}$ is the probability that the distributions of RMSd for the DT2 and ST2 models have the same mean. This p-value is sufficiently small to reject this hypothesis and to consider that the two distributions have significantly different means.

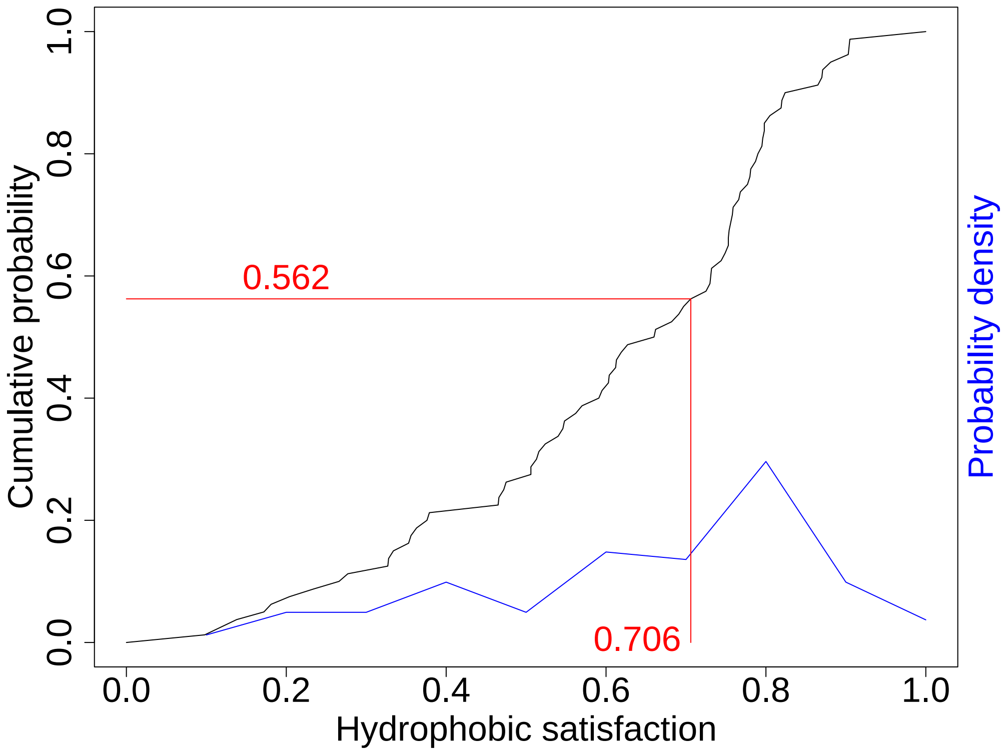

Figure S8: Density probability (blue line) and cumulative probability (black line) of the tryptophan hydrophobic satisfaction calculated with 79 all-α class protein 3D structures.
